# Evaporation Boundary Conditions for the Linear R13 Equations Based on the Onsager Theory

**DOI:** 10.3390/e20090680

**Published:** 2018-09-06

**Authors:** Alexander Felix Beckmann, Anirudh Singh Rana, Manuel Torrilhon, Henning Struchtrup

**Affiliations:** 1 Department of Mechanical Engineering, University of Victoria, Victoria, BC V8W 3P6, Canada; 2 Mathematics Institute, University of Warwick, Warwick CV4 7AL, UK; 3 Center for Computational Engineering Science (CCES), RWTH Aachen University, 52056 Aachen, Germany

**Keywords:** rarefied gas dynamics, modelling evaporation, R13-equations

## Abstract

Due to the failure of the continuum hypothesis for higher Knudsen numbers, rarefied gases and microflows of gases are particularly difficult to model. Macroscopic transport equations compete with particle methods, such as the Direct Simulation Monte Carlo method (DSMC), to find accurate solutions in the rarefied gas regime. Due to growing interest in micro flow applications, such as micro fuel cells, it is important to model and understand evaporation in this flow regime. Here, evaporation boundary conditions for the R13 equations, which are macroscopic transport equations with applicability in the rarefied gas regime, are derived. The new equations utilize Onsager relations, linear relations between thermodynamic fluxes and forces, with constant coefficients, that need to be determined. For this, the boundary conditions are fitted to DSMC data and compared to other R13 boundary conditions from kinetic theory and Navier–Stokes–Fourier (NSF) solutions for two one-dimensional steady-state problems. Overall, the suggested fittings of the new phenomenological boundary conditions show better agreement with DSMC than the alternative kinetic theory evaporation boundary conditions for R13. Furthermore, the new evaporation boundary conditions for R13 are implemented in a code for the numerical solution of complex, two-dimensional geometries and compared to NSF solutions. Different flow patterns between R13 and NSF for higher Knudsen numbers are observed.

## 1. Introduction

For modelling ideal gas flow, there are in general two approaches, the microscopic and the macroscopic approach. In the microscopic approach, the Boltzmann equation [1,2] is solved, e.g., with the Direct Simulation Monte Carlo method (DSMC) [3]. However, tracking particles is computationally expensive, and for engineering applications, determining the macroscopic quantities is often sufficient. In the macroscopic approach, microscopic information is condensed into quantities such as mass density, bulk velocity, temperature, heat flux and stress. Macroscopic transport equations reduce the number of variables and when simplified allow for analytical solutions. The advantage of faster calculations is associated with the restriction to certain flow regimes. Flow regimes can be characterized by the Knudsen number, which is the ratio of the mean free path, i.e., the average distance a molecule travels between two subsequent collisions, and a characteristic length, e.g., the diameter of a pipe. For Knudsen numbers larger than $\mathrm{Kn}\approx 4\times 10^{-2}$ [4], the classical Navier–Stokes–Fourier (NSF) equations start to fail [4,5]. Applications for Knudsen numbers in the transition regime, i.e., $4\times 10^{-2}<\mathrm{Kn}<2.5$ [4], may be those with large mean free paths, e.g., in vacuum or aerospace applications, or those with small characteristic lengths, which can be found in microflows. In this regime, rarefaction effects are observed, such as temperature jump and velocity slip at interfaces, Knudsen layers in front of interfaces, transpiration flow, thermal stresses or heat transfer without temperature gradients [4,5,6,7,8]. Knudsen layers are thin areas in front of boundaries in the order of a few mean free paths, where particle interaction with the boundary is the dominant mechanism.

By combining the Grad and Chapman–Enskog methods into the new order of magnitude method, Struchtrup and Torrilhon proposed the regularized R13 equations, macroscopic transport equations that account for effects in the transition regime [9]. Like all macroscopic transport equations, the R13 equations are an approximation of the Boltzmann equation. R13 introduces higher moments, which have a large influence in the rarefied gas regime and a small influence in the regime of small Knudsen numbers. Coefficients within the R13 equations allow quick adjustment between different collision models, such as Maxwell molecules, Hard-Spheres (HS) or the Bhatnagar–Gross–Krook (BGK) model [5]. In the following, only Maxwell molecules will be considered.

Due to increasing interest in Microelectromechanical devices (MEMS) [10], it is of interest to model evaporation processes for Knudsen numbers in the transition regime.

Based on microscopic boundary conditions of the Boltzmann equation, Struchtrup et al. derived macroscopic boundary conditions for R13 [11]. These equations, which are referred to as MBC (Macroscopic Boundary Conditions) in the following, show promising results for Knudsen numbers in the transition regime. Here, we seek to derive improved evaporation boundary conditions by using an entropy balance integrated around an interface between the liquid and vapour phase. Based on the Onsager theory, the integrated entropy balance is rewritten as the sum of thermodynamic fluxes and forces [12]. The Onsager theory assumes linear relations between fluxes and forces and allows one to break the entropy balance into sets of equations, which we utilize as evaporation/condensation boundary conditions [13,14].

A challenge lies in determining the Onsager coefficients, which provide the linear relations between fluxes and forces. The linear R13 equations, accompanied by the new Phenomenological Boundary Conditions (PBC), are solved for two one-dimensional, steady-state configurations. The first system consists of a vapour phase between two liquid reservoirs. A DSMC solution for this setup is used to fit the Onsager coefficients and to compare the results with the macroscopic boundary conditions for R13 and also with two Navier–Stokes–Fourier models, which are based on the Onsager theory as well. The second configuration is a half space problem [15], for which dimensionless flow parameters are used to compare the different models.

The remainder of the paper proceeds as follows: Section 1 gives an overview of the R13 equations and the corresponding macroscopic evaporation boundary conditions, based on kinetic theory. Section 2 explains the derivation of the Onsager boundary conditions. Section 3 shows how the Onsager coefficients are determined, mainly by fitting to DSMC data. In Section 4, the newly-derived boundary conditions are put to test in a numerical steady-state simulation with complex geometries. The work is summarized and discussed in Section 5.

### 1.1. The R13 Equations

In the following, all equations are non-dimensionalized and linearized around an equilibrium state defined by a reference density for the vapour $\rho_{0}$ and reference temperature $T_{0}$. The equilibrium saturation pressure for both liquid and vapour is defined as $p_{0}=p_{sat}\left(T_{0}\right)$. We shall consider small deviations from equilibrium, caused by pressure or temperature gradients, to drive evaporation or condensation. Non-dimensionalizing allows one to introduce meaningful coefficients into the equations, e.g., Prandtl or Knudsen numbers. The connection between variables denoting non-dimensional deviation to an equilibrium state (with hat) and the regular variables with dimension is:

(1) $$T=T_{0}\left(1+\hat{T}\right),\;\;\;\;\rho =\rho_{0}\left(1+\hat{\rho }\right),\;\;\;\;p=p_{0}\left(1+\hat{p}\right),$$

$$v_{k}=\sqrt{RT_{0}}{\hat{v}}_{k},\;\;\;\;q_{k}=\rho_{0}{\sqrt{RT_{o}}}^{3}{\hat{q}}_{k},\;\;\;\;\sigma_{ik}=\rho_{0}RT_{0}{\hat{\sigma }}_{ik},$$

$$h=h_{0}\left(1+\hat{h}\right),\;\;\;\;u=u_{0}\left(1+\hat{u}\right),\;\;\;\;\eta =\rho s=\eta_{0}\left(1+\hat{\eta }\right),$$

$$x_{k}=L{\hat{x}}_{k},\;\;\;t=\frac{L}{\sqrt{RT_{0}}}\hat{t}.$$

Here, *T* is temperature, ρ mass density, *p* pressure, $v_{k}$ the velocity vector, $q_{k}$ the heat flux vector, $\sigma_{ik}$ the stress tensor, *h* enthalpy, *u* internal energy, η=ρs entropy density, $x_{k}$ the position vector and *t* time. From now on, the hats are not shown.

The governing macroscopic equations that describe the gas are given by the conservation laws for mass, momentum and energy, which in linearized and dimensionless form read:

(2) $$\frac{\partial \rho }{\partial t}+\frac{\partial v_{k}}{\partial x_{k}}=0,$$

(3) $$\frac{\partial v_{i}}{\partial t}+\frac{\partial \sigma_{ik}}{\partial x_{k}}+\frac{\partial p}{\partial x_{i}}=F_{i},$$

(4) $$\frac{3}{2}\frac{\partial T}{\partial t}+\frac{\partial v_{k}}{\partial x_{k}}+\frac{\partial q_{k}}{\partial x_{k}}=0.$$

Here, $F_{i}$ is a body force, e.g., gravitational force. One has five equations for the five unknowns ρ, $v_{i}$ and *T*. An algebraic equation for *p* is found in the ideal gas law p=ρRT, which assumes for the non-dimensional and linear case the form p=ρ+T, with all variables describing the deviation to the equilibrium state.

It is necessary to find equations for the heat flux vector $q_{k}$ and stress tensor $\sigma_{ik}$, which beyond the hydrodynamic regime become full balance equations. By means of the order of magnitude method, Struchtrup and Torrilhon derived the following (here linearized and non-dimensionalized) balance equations from the Boltzmann equation, known as the regularized 13 moment equations [9],

(5) $$\frac{\partial \sigma_{ij}}{\partial t}+\frac{4}{5}\mathrm{Pr}\frac{{\bar{w}}_{3}}{{\bar{w}}_{2}}\frac{\partial q_{\langle i}}{\partial x_{j\rangle }}+\frac{\partial m_{ijk}}{\partial x_{k}}=-\frac{2}{{\bar{w}}_{2}}\frac{1}{\mathrm{Kn}}\left[\sigma_{ij}+2\mathrm{Kn}\frac{\partial v_{\langle i}}{\partial x_{j\rangle }}\right],$$

(6) $$\frac{\partial q_{i}}{\partial t}+\frac{5}{4\mathrm{Pr}}\frac{\theta_{4}}{\theta_{2}}\frac{\partial \sigma_{ik}}{\partial x_{k}}+\frac{1}{2}\frac{\partial R_{ik}}{\partial x_{k}}+\frac{1}{6}\frac{\partial \Delta }{\partial x_{i}}=-\frac{1}{\theta_{2}}\frac{5}{2\mathrm{Pr}}\frac{1}{\mathrm{Kn}}\left[q_{i}+\frac{5}{2\mathrm{Pr}}\mathrm{Kn}\frac{\partial T}{\partial x_{i}}\right].$$

The higher moments are defined over the relations:

(7) $$\Delta =-\frac{8\mathrm{Kn}}{{\mathrm{Pr}}_{\Delta }}\frac{\partial q_{k}}{\partial x_{k}},$$

(8) $$R_{ij}=-\frac{28}{5}\frac{\mathrm{Kn}}{{\mathrm{Pr}}_{R}}\frac{\partial q_{\langle i}}{\partial x_{j\rangle }},$$

(9) $$m_{ijk}=-\frac{3\mathrm{Kn}}{{\mathrm{Pr}}_{M}}\frac{\partial \sigma_{\langle ij}}{\partial x_{x\rangle }}.$$

By using the Chapman–Enskog expansion, while considering low Knudsen numbers, Equations (5) and (6) reduce to the laws of Navier–Stokes and Fourier, i.e., the left-hand sides become zero [5]. The balance laws (5) and (6) use the higher moments Δ, $R_{ik}$ and $m_{ijk}$. Here, $\mathrm{Pr}=\frac{\mu c_{p}}{k}$ denotes the Prandtl number, with μ as the shear viscosity. For a monatomic gas, one has $c_{p}=\frac{5}{2}R$ as the isobaric specific heat and $k=\frac{15}{4}\mu$ as the thermal conductivity. The Knudsen number is $\mathrm{Kn}=\frac{\mu \sqrt{RT}}{pL}$, with *L* as the characteristic length, e.g., the diameter of a pipe. Here, $\theta_{2}$, $\theta_{4}$, ${\bar{w}}_{2}$ and ${\bar{w}}_{3}$ are coefficients for different collision models, such as Maxwell, HS and BGK models. In the following sections, only Maxwell molecules are considered; nevertheless, the corresponding coefficients for Maxwell, HS or BGK models for stress tensor, heat flux vector and higher moments can be found in Table 1 [12].

### 1.2. Macroscopic Evaporation Boundary Conditions for Maxwell Molecules

For the case that a vapour molecule hitting the liquid interface is reflected back to the vapour and not being absorbed, Maxwell proposed an accommodation model, which is based on the assumption that the fraction χ of the vapour molecules hitting the liquid surface are diffusively reflected, i.e., with momentum and energy exchange, and the remaining fraction (1−χ) is specularly reflected, without energy exchange [7].

Based on microscopic evaporation boundary conditions of the Boltzmann equation, which are derived from a Maxwell model for the interface, Struchtrup et al. derived Macroscopic evaporation Boundary Conditions (MBC) for the R13 equations [11]. In these, interface effects are described through the accommodation coefficient χ and the evaporation coefficient ϑ. The evaporation coefficient equals the condensation coefficient, which is the probability that a vapour particle hitting the liquid interface will condense [16].

After non-dimensionalization and linearization around an equilibrium state, the MBC for evaporation [11] read:

(10) $$V_{n}=\sqrt{\frac{2}{\pi }}\frac{\vartheta }{2-\vartheta }\left(p_{sat}\left(T^{l}\right)-p^{g}+\frac{1}{2}\left(T^{g}-T^{l}\right)-\frac{1}{2}\sigma_{nn}^{g}+\frac{1}{120}\Delta +\frac{1}{28}R_{nn}\right),$$

(11) $$q_{n}^{g}=-\sqrt{\frac{2}{\pi }}\frac{\vartheta +\chi (1-\vartheta )}{2-\vartheta -\chi (1-\vartheta )}\left(2\left(T^{g}-T^{l}\right)+\frac{1}{2}\sigma_{nn}^{g}+\frac{1}{15}\Delta +\frac{5}{28}R_{nn}\right)-\frac{1}{2}V_{n}^{g},$$

(12) $$m_{nnn}=\sqrt{\frac{2}{\pi }}\frac{\vartheta +\chi (1-\vartheta )}{2-\vartheta -\chi (1-\vartheta )}\left(\frac{2}{5}\left(T^{g}-T^{l}\right)-\frac{7}{5}\sigma_{nn}^{g}+\frac{1}{75}\Delta -\frac{1}{14}R_{nn}\right)-\frac{2}{5}V_{n}^{g},$$

(13) $${\bar{\sigma }}_{nk}=-\sqrt{\frac{2}{\pi }}\frac{\vartheta +\chi (1-\vartheta )}{2-\vartheta -\chi (1-\vartheta )}\left({\bar{V}}_{k}^{g}+\frac{1}{5}{\bar{q}}_{k}^{g}+\frac{1}{2}{\bar{m}}_{nnk}\right),$$

(14) $${\bar{R}}_{nk}=\sqrt{\frac{2}{\pi }}\frac{\vartheta +\chi (1-\vartheta )}{2-\vartheta -\chi (1-\vartheta )}\left({\bar{V}}_{k}^{g}-\frac{11}{5}{\bar{q}}_{k}^{g}-\frac{1}{2}{\bar{m}}_{nnk}\right),$$

(15) $$\begin{array}{cc} {\tilde{m}}_{nij}=-\sqrt{\frac{2}{\pi }}\frac{\vartheta +\chi (1-\vartheta )}{2-\vartheta -\chi (1-\vartheta )} & \\ & \left({\tilde{\sigma }}_{ij}^{g}+\frac{1}{14}{\tilde{R}}_{ij}+\left(\frac{1}{5}\left(T^{g}-T^{l}\right)-\frac{1}{5}\sigma_{nn}^{g}+\frac{1}{150}\Delta \right)\delta_{ij}\right)+\frac{1}{5}\delta_{ij}V_{n}^{g}. \end{array}$$

Here, the index *n* refers to the direction normal to the interface. The Einstein notation, i.e., $A_{jj}=\sum_{j=1}^{3}A_{jj}$, is not applicable for the index *n*. The variables are tensor components, where the overbar denotes the normal-tangential and the tilde the tangential-tangential parts; see Appendix A. Note that all variables describe the deviation to an equilibrium state.

## 2. Evaporation Boundary Conditions for Linear R13 Based on the Second Law of Thermodynamics

The MBC have the major drawback of stability problems; see [17]. Therefore, we aim to derive stable Phenomenological Boundary Conditions (PBC) for the regularized R13 equations for a liquid-gas interface. The approach follows [12], in which a reduced entropy balance is used to derive boundary conditions for a wall-gas interface. The entropy balance for a fluid with dimensionless entropy density $\tilde{\eta }$, entropy flux $\Psi_{k}$ and entropy generation rate $\Sigma_{gen}$ reads:

(16) $$\frac{\partial \tilde{\eta }}{\partial t}+\frac{\partial \Psi_{k}}{\partial x_{k}}=\Sigma_{gen}.$$

Equation (16) shall be integrated over a small volume of area ΔA and height Δz across the liquid-vapour interface. By using Gauss’ theorem, the integrated entropy balance becomes:

(17) $$\int_{\Delta A\Delta z}\frac{\partial \tilde{\eta }}{\partial t}dV+\oint_{\partial \Delta V}\Psi_{k}n_{k}dA=\int_{\Delta A\Delta z}\Sigma_{gen}dV.$$

For Δz→0, the first term vanishes, and (17) reduces to the entropy balance for the interface,

(18) $$\left(\Psi_{k}^{g}-\Psi_{k}^{l}\right)n_{k}=\Sigma_{surface}\geq 0.$$

Hence, the entropy generation rate $\Sigma_{surface}=\frac{1}{dA}\int_{\Delta A\Delta z}\Sigma_{gen}dV$ is equal to the difference in entropy fluxes entering and leaving the interface. In the following, all variables on the liquid side are denoted with *l* and all variables on the vapour side with *g*. A linear combination of manipulated mass, energy and entropy balances (Appendix B) leads to the (linearized and non-dimensional) entropy flux on the liquid side as:

(19) $$\Psi_{k}^{l}=-q_{k}^{l}T^{l}-\sigma_{ik}^{l}v_{i}^{l}-p^{l}v_{k}^{l}.$$

Here, *T*, ρ and *v* are deviations from an equilibrium state defined by $T_{0}$, $\rho_{0}$ and $p_{0}=p_{sat}\left(T_{0}\right)$. For the linear R13 equations and the vapour side, the linearized and dimensionless entropy flux (Appendix B) is:

(20) $$\Psi_{k}^{g}=-\left(\rho^{g}+T^{g}\right)v_{k}^{g}-v_{i}^{g}\sigma_{ik}^{g}-T^{g}q_{k}^{g}-\frac{\varpi_{3}}{5}\mathrm{Pr}q_{i}^{g}\sigma_{ik}^{g}-\frac{\varpi_{2}}{4}\sigma_{ij}^{g}m_{ijk}-\frac{2\theta_{2}}{25}{\left(\mathrm{Pr}\right)}^{2}\left(q_{i}^{g}R_{ik}+\frac{\Delta }{3}q_{k}^{g}\right).$$

Furthermore, the (linearized and non-dimensional) balance laws for mass, momentum and energy, integrated around the interface similar to (18), become:

(21) $$\rho_{l}v_{k}^{l}n_{k}=\rho_{0}v_{k}^{g}n_{k},$$

(22) $$p^{l}n_{i}+\sigma_{ik}^{l}n_{k}=p^{g}n_{i}+\sigma_{ik}^{g}n_{k},$$

(23) $$\frac{\rho_{l}h_{0}^{l}}{R\rho_{0}T_{0}}v_{k}^{l}n_{k}+q_{k}^{l}n_{k}=\frac{h_{o}^{g}}{RT_{o}}v_{k}^{g}n_{k}+q_{k}^{g}n_{k}.$$

The variables $v_{k}^{l}$ and $v_{k}^{g}$ are the velocities on the liquid and vapour sides from the perspective of an observer resting on the interface.

The entropy fluxes (19) and (20) are plugged into the integrated entropy balance (18). Equations (21)–(23) are used to eliminate the variables $v_{k}^{l}$, $\sigma_{ik}^{l}$ and $q_{k}^{l}$. All variables describe the deviation to equilibrium, are dimensionless and linearized. After applying the appropriate coefficients for Maxwell molecules, according to Table 1, using the Clausius–Clapeyron equation [18] (linearized and dimensionless) in the form $p_{sat}\left(T^{l}\right)=\frac{h_{gl}^{0}}{RT_{0}}T^{l}$ and by considering $\rho_{l}\gg \rho_{0}$, one may write (18) as:

(24) $$\begin{array}{cc} J_{k}^{g}n_{k}\frac{1}{\rho_{0}}\left(p_{sat}\left(T^{l}\right)-p^{g}\right)- & \left(T^{g}-T^{l}\right)q_{k}^{g}n_{k}-V_{i}\sigma_{ik}^{g}n_{k}-\frac{\varpi_{3}}{5}\mathrm{Pr}q_{i}^{g}\sigma_{ik}^{g}n_{k}\\ & \;-\frac{\varpi_{2}}{4}\sigma_{ij}^{g}m_{ijk}n_{k}-\frac{2\theta_{2}}{25}{\left(\mathrm{Pr}\right)}^{2}\left(q_{i}^{g}R_{ik}n_{k}+\frac{\Delta }{3}q_{k}^{g}n_{k}\right)=\Sigma_{surface}\geq 0, \end{array}$$

where $V_{i}=v_{i}^{g}-v_{i}^{l}$, $J_{k}^{g}n_{k}=\rho_{0}v_{k}^{g}n_{k}$ and the corresponding ideal gas law, given as $\rho^{g}=p^{g}-T^{g}$, was used. To accomplish a proper entropy balance for the linearized equations, terms up to second order are kept [19].

Next, the entropy balance is split into contributions from normal and tangential components (see Appendix A); all matrices and higher moments are symmetric and trace free,

(25) $$\begin{array}{cc} \Sigma_{surface} & =J_{n}^{g}\frac{1}{\rho_{0}}\left[p_{sat}\left(T^{l}\right)-p^{g}-\sigma_{nn}\right]\\ & +q_{n}^{g}\left[-\left(T^{g}-T^{l}\right)-\frac{\varpi_{3}}{5}\mathrm{Pr}\sigma_{nn}-\frac{2\theta_{2}}{25}{\left(\mathrm{Pr}\right)}^{2}\left(R_{nn}+\frac{\Delta }{3}\right)\right]\\ & +m_{nnn}\left[-\frac{3\varpi_{2}}{8}\sigma_{nn}\right]\\ & +{\bar{\sigma }}_{nk}\left[-{\bar{V}}_{k}-\frac{\varpi_{3}}{5}\mathrm{Pr}{\bar{q}}_{k}-\frac{\varpi_{2}}{2}{\bar{m}}_{nnk}\right]+{\bar{R}}_{nk}\left[-\frac{2\theta_{2}}{25}{\left(\mathrm{Pr}\right)}^{2}{\bar{q}}_{k}\right]\\ & +{\tilde{m}}_{nij}\left[-\frac{\varpi_{2}}{4}{\tilde{\sigma }}_{ij}\right]. \end{array}$$

As before, the overbar denotes normal-tangential, and the tilde denotes tangential-tangential components. In the case that the mass flow $J_{n}^{g}$ vanishes, Equation (25) simplifies to the entropy generation at a wall-gas-interface; see [12].

The entropy generation may be written as a superposition of thermodynamic fluxes $J_{i}$ and forces $X_{i}$ [13,14]:

(26) $$\Sigma_{surface}=\sum_{i}J_{i}X_{i}\geq 0.$$

Here, moments with odd degree in the normal direction *n* are identified as fluxes, i.e., $J_{n}$, $q_{n}$, $m_{nnn}$, ${\bar{\sigma }}_{nk}$, ${\bar{R}}_{nk}$ and ${\tilde{m}}_{nij}$, while moments with even degree in *n* are identified as the corresponding forces, i.e., $p^{g}$, $T^{g}$, $T^{l}$, $\sigma_{nn}$, $R_{nn}$, Δ, ${\bar{V}}_{k}$, ${\bar{q}}_{k}$, ${\bar{m}}_{nnk}$ and ${\tilde{\sigma }}_{ij}$. Note that $p^{g}$, $T^{g}$, $T^{l}$, $\sigma_{nn}$, $R_{nn}$, Δ, $J_{n}$, $q_{n}$ and $m_{nnn}$ are scalars, ${\bar{V}}_{k}$, ${\bar{q}}_{k}$, ${\bar{m}}_{nnk}$, ${\bar{\sigma }}_{nk}$ and ${\bar{R}}_{nk}$ are vectors and ${\tilde{\sigma }}_{ij}$ and ${\tilde{m}}_{nij}$ are tensors. Furthermore, a linear force-flux relation is stated within the Onsager theory, to satisfy Equation (26):

(27) $$J_{i}=\sum_{j}L_{ij}X_{j}.$$

Here, $L_{ij}$ is a positive-definite matrix of Onsager coefficients with the Onsager reciprocity relation, requiring symmetry of $L_{ij}$. Only equations of the same tensor rank are coupled over the reciprocity relation (Curie principle [20]). This means that all force terms of the same tensor rank superimpose on each other and impact all fluxes of the same tensor rank; hence:

Scalar fluxes:

(28) $$\left(\begin{array}{c} V_{n}^{g}\\ q_{n}^{g}\\ m_{nnn} \end{array}\right)=\left(\begin{array}{ccc} \lambda_{0} & \lambda_{1} & \lambda_{2}\\ \lambda_{1} & \lambda_{3} & \lambda_{4}\\ \lambda_{2} & \lambda_{4} & \lambda_{5} \end{array}\right)\left(\begin{array}{c} \left[p_{sat}\left(T^{l}\right)-p^{g}-\sigma_{nn}\right]\\ \left[-\left(T^{g}-T^{l}\right)-\frac{\varpi_{3}}{5}\mathrm{Pr}\sigma_{nn}-\frac{2\theta_{2}}{25}{\left(\mathrm{Pr}\right)}^{2}\left(R_{nn}+\frac{\Delta }{3}\right)\right]\\ \left[-\frac{3\varpi_{2}}{8}\sigma_{nn}\right] \end{array}\right)$$

Vector fluxes:

(29) $$\left(\begin{array}{c} {\bar{\sigma }}_{nk}\\ {\bar{R}}_{nk} \end{array}\right)=\left(\begin{array}{cc} \zeta_{0} & \zeta_{1}\\ \zeta_{1} & \zeta_{2} \end{array}\right)\left(\begin{array}{c} \left[-{\bar{V}}_{k}-\frac{\varpi_{3}}{5}\mathrm{Pr}{\bar{q}}_{k}-\frac{\varpi_{2}}{2}{\bar{m}}_{nnk}\right]\\ \left[-\frac{2\theta_{2}}{25}{\left(\mathrm{Pr}\right)}^{2}{\bar{q}}_{k}\right] \end{array}\right)$$

Tensor fluxes:

(30) $${\tilde{m}}_{nij}=-\kappa_{0}\frac{\varpi_{2}}{4}{\tilde{\sigma }}_{ij}$$

For $\lambda_{0}=\lambda_{1}=\lambda_{2}=0$, one obtains the full set of phenomenological boundary conditions for a wall-gas interface, which are independent of evaporation as in [12]. The interface conditions (29) and (30), which consist of first order tensors (vectors) and second order tensors (matrices), respectively, have been fitted for a wall-gas interface in [12]. The fitting of (28) for evaporation at liquid-vapour interfaces shall be discussed in Section 3. In the following, the new evaporation boundary conditions (28)–(30) shall be referred to as PBC.

## 3. Determining the Onsager Coefficients

### 3.1. Comparison to Previous Macroscopic Boundary Conditions

The structure of PBC and MBC is very similar; the main difference lies in the values of the coefficients. As a first step for determining the Onsager coefficients of the PBC (28)–(30), we aim to use the coefficients of the MBC in a way that all terms, except those where higher order moments, i.e., Δ, $R_{ij}$, $m_{ijk}$, occur, agree with the MBC. This is justified due to the fact that the MBC predict effects in the Navier–Stokes regime very well. In the rarefied gas regime, however, their application seems to be more limited [11]. Since the higher moments are responsible for predicting a simplified Knudsen layer and also for rarefaction effects, a difference between PBC and MBC in these terms is desired. For a liquid-gas interface, the matrix of Onsager coefficients of those boundary conditions with variables of zero tensor rank (28) assumes the dimension 3 × 3, in contrast to the wall-gas interface, where the matrix reads 2 × 2 [12]. Based on these thoughts, the following Onsager coefficients are suggested:

(31) $$\lambda_{0}=a\vartheta_{2},$$

(32) $$\lambda_{1}=b\left(-\frac{1}{2}\vartheta_{2}\right),$$

(33) $$\lambda_{2}=c\left(-\frac{2}{5}\vartheta_{2}\right),$$

(34) $$\lambda_{3}=d\left(\frac{1}{4}\vartheta_{2}+2\chi_{2}\right),$$

(35) $$\lambda_{4}=e\left(\frac{1}{5}\vartheta_{2}-\frac{2}{5}\chi_{2}\right),$$

(36) $$\lambda_{5}=f\left(\frac{4}{25}\vartheta_{2}+\frac{52}{25}\chi_{2}\right),$$

with:

$$\vartheta_{2}=\sqrt{\frac{2}{\pi }}\frac{\vartheta }{2-\vartheta }\;,\;\;\;\;\;\;\;\chi_{2}=\sqrt{\frac{2}{\pi }}\frac{\vartheta +\chi (1-\vartheta )}{2-\vartheta -\chi (1-\vartheta )}.$$

To leave the coefficients adjustable, the factors a…f have been introduced. For a=b=…=f=1, the PBC differ from the MBC, only in the higher order terms; see Appendix C. The boundary conditions (29) and (30) have been fitted for a wall-gas interface in [12] and shall not further be investigated here. To determine the coefficients a…f by fitting to a DSMC solution, two evaporation problems will be discussed, for which analytical solutions for R13 with PBC can be obtained.

### 3.2. Simplification of R13 for 1D Problems

As can be expected, the present PBC, just like the MBC, give less accurate results than methods that solve the full Boltzmann equation. The R13 equations and their corresponding interface and boundary conditions are simplifications of the Boltzmann equation and carry less information. The adjustable coefficients a…f in (31)–(36) leave six degrees of freedom to determine the Onsager coefficients. It is of interest whether the simplification of R13 to the Boltzmann equation can be partly corrected by adjusting the Onsager coefficients. In this context, we simplify the linear R13 equations for one-dimensional and steady systems and solve them for two problems, previously discussed in [11]. Then, the new solutions are fitted to DSMC data.

All variables depend only on the location *x*. For the equilibrium rest state, the saturation pressure of the liquid interface is set to $p_{sat}\left(T_{0}\right)=p_{0}$. We assume that the liquid temperature at the interface is controlled. Small pressure or temperature changes are sufficient to drive evaporation or condensation. All equations are linear and dimensionless and describe the deviation to their equilibrium state. The simplified balance equations for mass, momentum and energy read:

(37) $$\frac{\partial v}{\partial x}=\frac{\partial \sigma }{\partial x}+\frac{\partial p}{\partial x}=\frac{\partial q}{\partial x}=0.$$

After, simple integration follows:

(38) $$v=V_{0}=const,\;\;\;\;\;p+\sigma =P_{0}=const,\;\;\;\;\;q_{0}=Q_{0}=const.$$

Hence, velocity and conductive heat flux are constant in the vapour phase. The normal components of the linear and non-dimensional constitutive equations for (7)–(9) obtain the form:

(39) $$\Delta =-\frac{8\mathrm{Kn}}{{\mathrm{Pr}}_{\Delta }}\frac{\partial q}{\partial x}=0,\;\;\;\;\;R_{nn}=-\frac{28}{5}\frac{\mathrm{Kn}}{{\mathrm{Pr}}_{R}}\frac{\partial q}{\partial x}=0,\;\;\;\;\;m_{nnn}=-\frac{3\mathrm{Kn}}{{\mathrm{Pr}}_{M}}\frac{\partial \sigma }{\partial x},$$

with data to adjust between the molecule models from Table 1. The linear and non-dimensional equations for normal stress σ and conductive heat flux $q_{o}$ become:

(40) $$\frac{6}{5}\mathrm{Kn}\frac{\partial^{2}\sigma }{\partial x^{2}}=\frac{\sigma }{\mathrm{Kn}},$$

(41) $$\frac{\partial T_{g}}{\partial x}=-\frac{4q_{0}}{15\mathrm{Kn}}-\frac{2}{5}\frac{\partial \sigma }{\partial x}.$$

Integration yields:

(42) $$\sigma =A\sinh\left[\sqrt{\frac{5}{6}}\frac{x}{\mathrm{Kn}}\right]+B\cosh\left[\sqrt{\frac{5}{6}}\frac{x}{\mathrm{Kn}}\right],$$

(43) $$T_{g}=K-\frac{4q_{0}x}{15\mathrm{Kn}}-\frac{2}{5}\sigma ,$$

with *A*, *B*, *K* as constants of integration. There are six unknowns ($V_{0}$, $P_{0}$, $Q_{0}$, *A*, *B*, *K*), that must be determined for finding the solution. For evaporating interfaces and by taking Δ=R=0 (39) into account, the normal boundary conditions (28) simplify to:

(44) $$V_{o}=\lambda_{0}\left[-P_{0}+p_{sat}\left(T^{l}\right)\right]+\lambda_{1}\left[-\left(T_{g}-T_{l}\right)-\frac{\varpi_{3}}{5}\mathrm{Pr}\sigma \right]-\lambda_{2}\frac{3\varpi_{2}}{8}\sigma ,$$

(45) $$q_{o}=\lambda_{1}\left[-P_{0}+p_{sat}\left(T^{l}\right)\right]+\lambda_{3}\left[-\left(T_{g}-T_{l}\right)-\frac{\varpi_{3}}{5}\mathrm{Pr}\sigma \right]-\lambda_{4}\frac{3\varpi_{2}}{8}\sigma ,$$

(46) $$\frac{6}{5}\mathrm{Kn}\left[\frac{\partial \sigma }{\partial x}\right]=\lambda_{2}\left[P_{0}-p_{sat}\left(T^{l}\right)\right]+\lambda_{4}\left[\left(T_{g}-T_{l}\right)+\frac{\varpi_{3}}{5}\mathrm{Pr}\sigma \right]+\lambda_{5}\frac{3\varpi_{2}}{8}\sigma ,$$

with $V_{o}=n_{k}V_{k}$ and $q_{o}=q_{k}n_{k}$.

### 3.3. Problem I: Vapour Layer between Two Liquid Reservoirs

**Figure 1:**
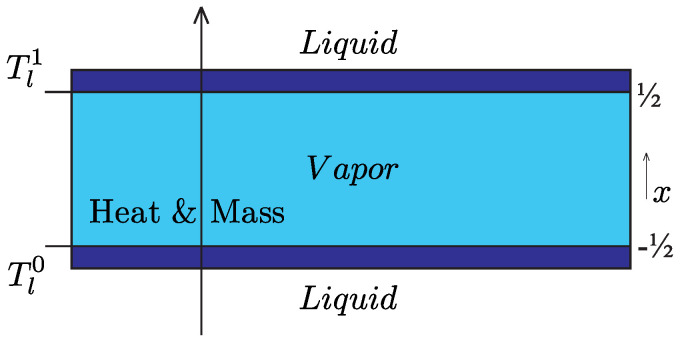
System I: Vapour phase between two liquid reservoirs.

In the first problem for fitting the coefficients a…f and also for gaining insight into the Knudsen layers, we consider one-dimensional, steady-state heat and mass transfer within a vapour phase in between two liquid reservoirs with controlled temperature on the liquid side of the liquid-vapour interfaces. The configuration shown in Figure 1 has been discussed in [11] and shall be outlined only briefly here.

The interfaces are located at $x=\pm \frac{1}{2}$ with the normal vector *n* pointing from liquid into vapour and the superscripts 0 for $x=-\frac{1}{2}$ and 1 for $x=\frac{1}{2}$, i.e., $V_{0}^{0}=-V_{0}^{1}=V_{0}$. The driving force for evaporation and condensation is the temperature difference between $T_{l}^{0}$ and $T_{l}^{1}$. The required six equations are found by evaluating the boundary conditions (28) at both interfaces. For evaluation of the equations, it is convenient to take both the sums and the differences at both interfaces. For the three sums, it follows:

(47) $$P_{o}=\frac{1}{2}\left(p_{sat}^{0}\left(T_{l}^{0}\right)+p_{sat}^{0}\left(T_{l}^{1}\right)\right),$$

(48) $$\left(T_{l}^{0}+T_{l}^{1}\right)-\left(T_{g}^{0}+T_{g}^{1}\right)=0,$$

(49) $$\sigma^{0}=-\sigma^{1}.$$

Stress profile Equation (42) and temperature profile Equation (43) follow as:

(50) $$\sigma =A\sinh\left[\sqrt{\frac{5}{6}}\frac{x}{\mathrm{Kn}}\right],$$

(51) $$T_{g}=\frac{\left(T_{l}^{0}+T_{l}^{1}\right)}{2}-\frac{4q_{0}x}{15\mathrm{Kn}}-\frac{2}{5}A\sinh\left[\sqrt{\frac{5}{6}}\frac{x}{\mathrm{Kn}}\right].$$

The three differences of the normal boundary conditions form a linear system for $V_{0}$, $Q_{0}$ and *A* as:

(52) $$V_{0}=\frac{1}{2}\left(\begin{array}{c} \lambda_{0}\left[p_{sat}\left(T_{l}^{0}\right)-p_{sat}\left(T_{l}^{1}\right)\right]\\ +\lambda_{1}\left[-\frac{4q_{0}}{15\mathrm{Kn}}+\left(T_{l}^{0}-T_{l}^{1}\right)+\left(\frac{2\varpi_{3}}{5}\mathrm{Pr}-\frac{4}{5}\right)A\sinh\left[\frac{1}{2}\sqrt{\frac{5}{6}}\frac{1}{\mathrm{Kn}}\right]\right]\\ +\frac{3\varpi_{2}}{4}\lambda_{2}A\sinh\left[\frac{1}{2}\sqrt{\frac{5}{6}}\frac{1}{\mathrm{Kn}}\right] \end{array}\right),$$

(53) $$Q_{0}=\frac{1}{2}\left(\begin{array}{c} \lambda_{1}\left[p_{sat}\left(T_{l}^{0}\right)-p_{sat}\left(T_{l}^{1}\right)\right]\\ +\lambda_{3}\left[-\frac{4q_{0}}{15\mathrm{Kn}}+\left(T_{l}^{0}-T_{l}^{1}\right)+\left(\frac{2\varpi_{3}}{5}\mathrm{Pr}-\frac{4}{5}\right)A\sinh\left[\frac{1}{2}\sqrt{\frac{5}{6}}\frac{1}{\mathrm{Kn}}\right]\right]\\ +\lambda_{4}\frac{3\varpi_{2}}{4}A\sinh\left[\frac{1}{2}\sqrt{\frac{5}{6}}\frac{1}{\mathrm{Kn}}\right] \end{array}\right),$$

(54) $$\begin{array}{cc} A=\frac{1}{\frac{12}{5}\sqrt{\frac{5}{6}}\cosh\left(\frac{1}{2}\sqrt{\frac{5}{6}}\frac{1}{\mathrm{Kn}}\right)} & \\ & \left(\begin{array}{c} \lambda_{4}\left[\frac{4q_{o}}{15\mathrm{Kn}}+\left(T_{l}^{1}-T_{l}^{0}\right)+\left(\frac{4}{5}-\frac{2\varpi_{3}}{5}\mathrm{Pr}\right)A\sinh\left[\frac{1}{2}\sqrt{\frac{5}{6}}\frac{1}{\mathrm{Kn}}\right]\right]\\ -\lambda_{5}\frac{3\varpi_{2}}{4}A\sinh\left[\frac{1}{2}\sqrt{\frac{5}{6}}\frac{1}{\mathrm{Kn}}\right]+\lambda_{2}\left[p_{sat}\left(T_{l}^{1}\right)-p_{sat}\left(T_{l}^{0}\right)\right] \end{array}\right). \end{array}$$

Here, *A* is the amplitude of the Knudsen layer. We refrain from showing the solution, and will only show results from the inversion in the figures. For the linear NSF-Onsager boundary conditions (see Appendix D), one finds:

(55) $$V_{0}=\frac{r_{22}}{r_{11}r_{22}-r_{12}r_{12}}\frac{1}{\sqrt{2\pi }}\frac{1}{2}\left(p_{sat}^{0}\left(T_{l}^{0}\right)-p_{sat}^{1}\left(T_{l}^{1}\right)+\frac{r_{12}}{r_{22}}\left(\frac{4Q_{0}}{15\mathrm{Kn}}+T_{l}^{1}-T_{l}^{0}\right)\right),$$

(56) $$q_{0}=\frac{1}{r_{22}}\frac{1}{2}\left(\frac{1}{\sqrt{2\pi }}\left(-\frac{4Q_{0}}{15\mathrm{Kn}}+T_{l}^{0}-T_{l}^{1}\right)-2r_{12}V_{0}\right),\;\;\;\;\;\;\;A=0.$$

The given solution for NSF is a simplification for χ=ϑ=1; see Appendix D. For the NSF-Onsager coefficients $r_{11}$, $r_{12}$ and $r_{22}$, the Onsager matrix (A30) or the corrected Onsager matrix (A31) can be used. The solution of the MBC for this system can be found in [11]. Results shall be compared in Section 3.5 and Section 3.6.

### 3.4. Problem II: Evaporation in the Half-Space Problem

**Figure 2:**
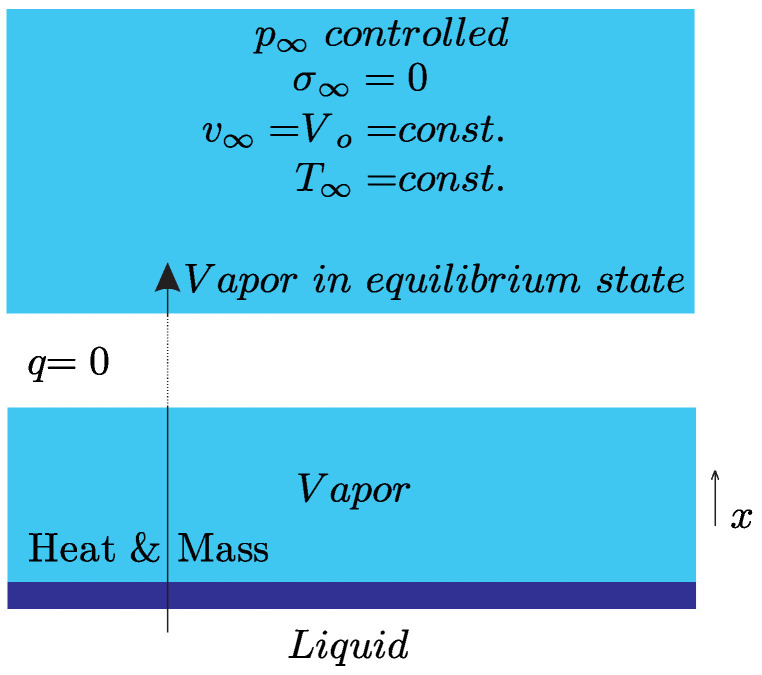
System II: Half-space problem.

In the half space problem, a liquid interface evaporates into the equilibrium state, as discussed previously in [11]. The driving force is the prescribed pressure $p_{\infty }$ far away from the interface; see Figure 2.

The six unknowns are found by considering evaporation boundary conditions on one side and constant velocity $v_{\infty }=V_{0}$, pressure $p_{\infty }=P_{0}$ and temperature $T_{\infty }$ far away from the interface. For reaching constant pressure $p_{\infty }$ and due to the momentum balance (38), it is necessary to set the normal stress far away from the interface to $\sigma_{\infty }=0$. Moreover, conductive heat flux $q_{0}$ is set to zero, as well. With $T_{\infty }$ prescribed, one finds the constant *K*. For (50) and (51), it follows:

(57) $$\sigma \left(x\right)=A\exp\left[-\sqrt{\frac{5}{6}}\frac{x}{\mathrm{Kn}}\right],$$

(58) $$T\left(x\right)=T_{\infty }-\frac{2}{5}\sigma \left(x\right).$$

Evaluating the boundary conditions (28) at the interface between liquid and vapour leads to:

(59) $$v_{\infty }=\lambda_{0}\left[p_{sat}\left(T_{l}\right)-p_{\infty }\right]+\lambda_{1}\left(T_{l}-T_{\infty }\right)+\left(\lambda_{1}\left(\frac{2}{5}-\frac{\varpi_{3}}{5}\mathrm{Pr}\right)-\lambda_{2}\frac{3\varpi_{2}}{8}\right)A,$$

(60) $$0=\lambda_{1}\left[p_{sat}\left(T_{l}\right)-p_{\infty }\right]+\lambda_{3}\left(T_{l}-T_{\infty }\right)+\left(\lambda_{3}\left(\frac{2}{5}-\frac{\varpi_{3}}{5}\mathrm{Pr}\right)-\lambda_{4}\frac{3\varpi_{2}}{8}\right)A,$$

(61) $$0=\lambda_{2}\left[p_{sat}\left(T_{l}\right)-p_{\infty }\right]+\lambda_{4}\left(T_{l}-T_{\infty }\right)+\left(\lambda_{4}\left(\frac{2}{5}-\frac{\varpi_{3}}{5}\mathrm{Pr}\right)-\lambda_{5}\frac{3\varpi_{2}}{8}-\frac{6}{5}\sqrt{\frac{5}{6}}\right)A.$$

For the Navier–Stokes–Fourier equation out of Equation (A29), it follows:

(62) $$v_{\infty }=\frac{p_{sat}\left(T_{l}\right)-p_{\infty }}{\sqrt{2\pi }r_{11}},$$

(63) $$v_{\infty }=\frac{1}{\sqrt{2\pi }}\frac{T_{l}-T_{\infty }}{r_{21}}.$$

With prescribed pressure $p_{\infty }$ and by setting $p_{sat}\left(T_{l}\right)-p_{\infty }=\Delta p$ and $T_{l}-T_{\infty }=\Delta T$, there are three unknowns $v_{\infty }$, $T_{\infty }$ and *A*, which can be calculated with (59)–(61) for PBC and (62) and (63) for NSF. The solution for the MBC can again be found in [11]. Note that for NSF, *A* is zero, and the two given equations are sufficient.

Ytrehus, who discussed the half space problem in [15], proposed dimensionless ratios in which the prescribed pressure $p_{\infty }$ is eliminated. The ratios that make it easy to compare different models, e.g., Maxwell molecules, BGK, Navier–Stokes–Fourier, etc., read:

(64) $$\alpha_{p}=\frac{p_{sat}\left(T_{l}\right)-p_{\infty }}{\frac{v_{\infty }}{\sqrt{2}}},$$

(65) $$\alpha_{\theta }=\frac{T_{l}-T_{\infty }}{\frac{v_{\infty }}{\sqrt{2}}}.$$

Note that (59)–(63) and therefore also (64) and (65) are independent of the Knudsen number.

### 3.5. Fitting of the Onsager Coefficients: Standard Temperature Profile

The ratios (64) and (65) from Problem II together with DSMC data for Problem I shall be used to fit the coefficients a…f in (31)–(36). The temperatures and saturation pressures at the liquid boundaries are given as $T_{l}^{0}=p_{sat}\left(T_{l}^{0}\right)=1.05$ and $T_{l}^{1}=p_{sat}\left(T_{l}^{1}\right)=0.95$. All results in the following are based on full evaporation and fully-diffusive reflection, by setting the evaporation and accommodation coefficients ϑ=χ=1. Maxwell molecules are considered, and their data are taken out of Table 1. In Table 2, factors for the Onsager coefficients, used in Equations (31)–(36), which have been found by trial and error, are suggested to adjust the PBC, Equations (28), for the best fit. The results of the new PBC are compared with the previously derived evaporation boundary conditions (MBC) and also with Navier–Stokes–Fourier solutions. NSF is based on Onsager boundary conditions, as well, and uses the Onsager matrix (A30) or the corrected Onsager matrix (A31).

Ytrehus used a moment method to solve the half space problem with high precision [15] and his results are used here as a reference. Ytrehus’ ratios $\alpha_{p}$, $\alpha_{\theta }$ (64) and (65) have been calculated for PBC, MBC, NSF and corrected NSF. Together with the percentual deviation to Ytrehus’ solution, they are given in Table 3.

By trial and error fitting of the Onsager coefficients, it was not possible to achieve superior agreement between PBC and DSMC for Problem I (Section 3.3) and proper results for Ytrehus’ ratios (64) and (65) at the same time. Forcing good agreement between Ytrehus’ solution of the half space problem and PBC regarding the dimensionless ratios showed a significant decrease in agreement between PBC and DSMC for Problem I. The fittings that are chosen here are compromises between Problem I and Problem II, but with strong emphasis on achieving proper results for Problem I, which means proper agreement with DSMC results.

**Figure 3:**
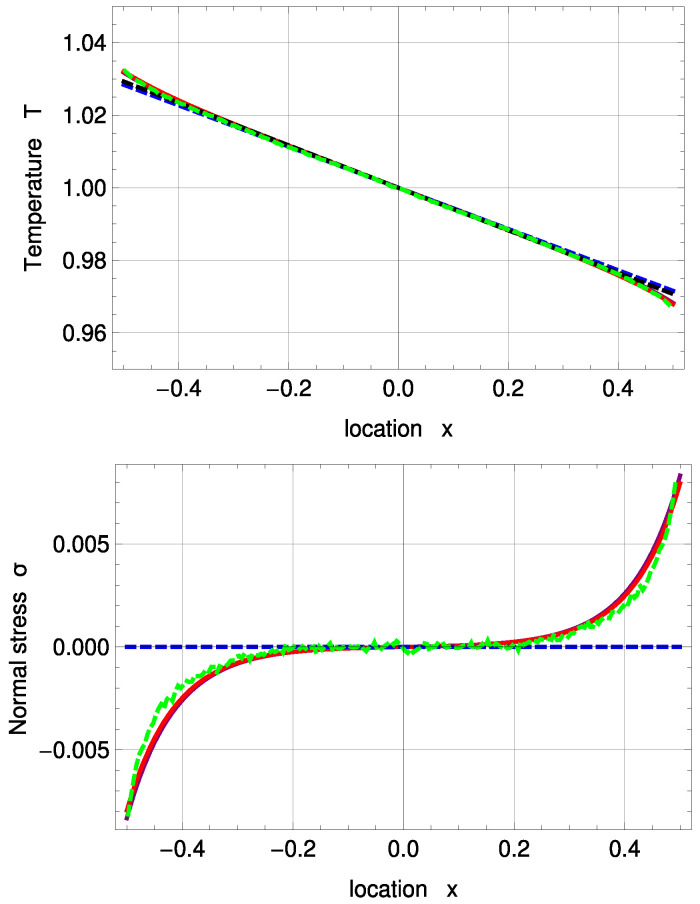
Temperature and normal stress profiles for Kn=0.078 with ΔT=0.05 and Δp=0.05: Direct Simulation Monte Carlo method (DSMC) (symmetrized; green, dashed), R13 with PBC (purple), R13 with MBC (red), corrected NSF (blue, dashed), uncorrected NSF (black, dashed). Note: In the upper plot, all curves superimpose on each other. In the lower plot, both NSF models are zero.

Figure 3 shows temperature and normal stress profiles for Kn=0.078. R13 with PBC (solid, purple) and MBC (solid, red) are in good agreement with DSMC (green, dashed). The amplitude of the Knudsen layer *A* is zero for NSF (black, dashed) and corrected NSF (blue, dashed). As a result, both NSF solutions slightly deviate from DSMC close to the boundaries. A=0 removes the last term in (51) and therefore leads to a linear function. In Problem I, NSF is not able to predict normal stress at all; see Equations (55) and (56).

**Figure 4:**
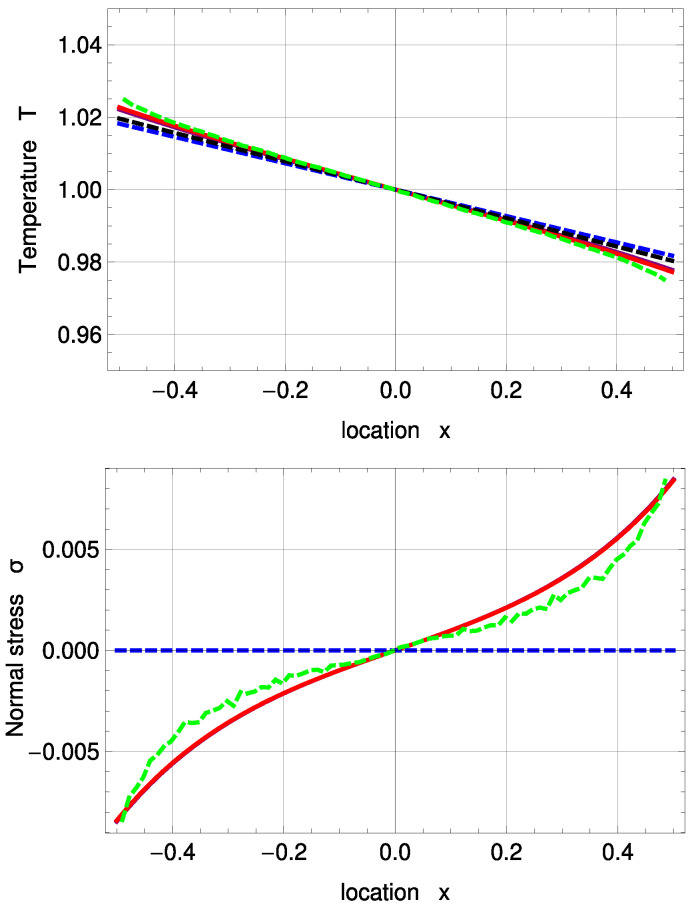
Temperature and normal stress profiles for Kn=0.235 with ΔT=0.05 and Δp=0.05: DSMC (symmetrized; green, dashed), R13 with PBC (purple), R13 with MBC (red), corrected NSF (blue, dashed), uncorrected NSF (black, dashed). Note: In the upper plot, all curves superimpose on each other. In the lower plot, both NSF models are zero.

In Figure 4, temperature and normal stress profiles are illustrated for Kn=0.235. Both sets of boundary conditions for R13 reconstruct the DSMC results well, but slightly underpredict the Knudsen layers both for temperature and normal stress. For the temperature profile, they are in better agreement with DSMC than the two NSF solutions. For both Kn=0.078 and Kn=0.235, one notes the significant temperature jumps at the boundaries.

**Figure 5:**
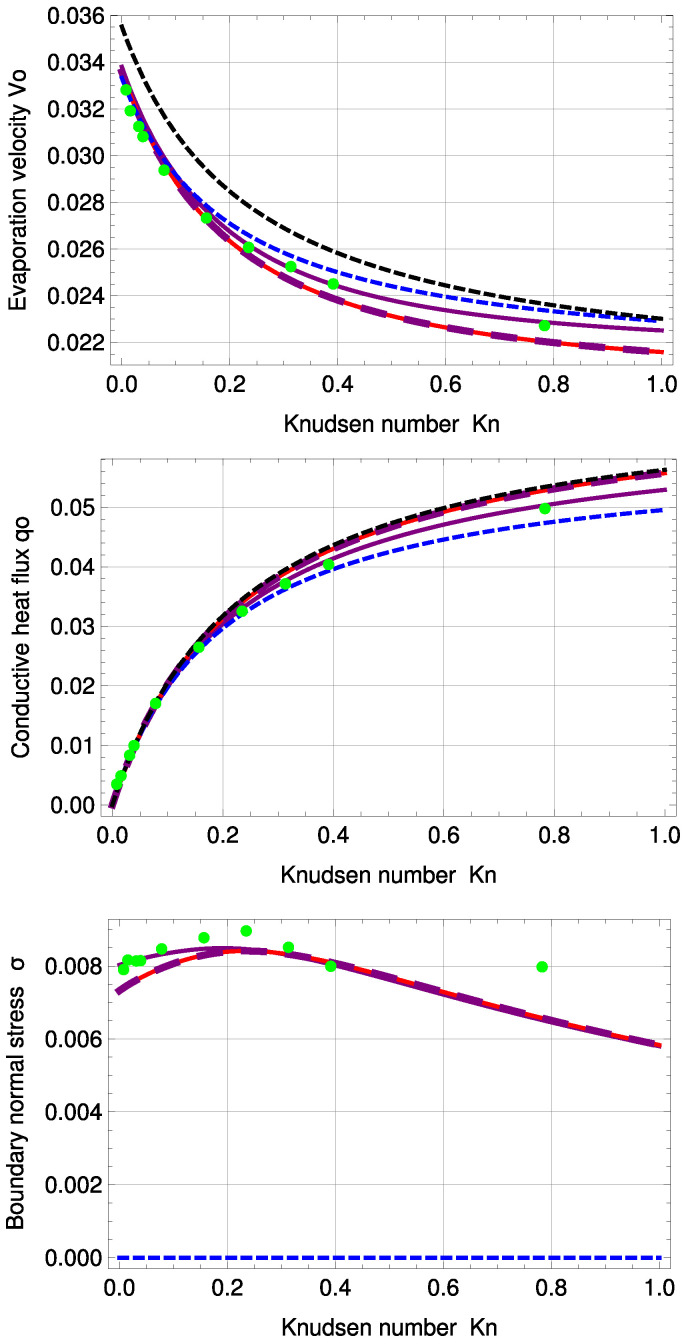
Evaporation velocity $V_{0}$, conductive heat flux $q_{0}$ and boundary normal stress $\sigma_{0}$ for standard temperature profile: DSMC (green, dots), R13 with PBC (purple), R13 with PBC: a…f=1 (purple, large, dashed), R13 with MBC (red), corrected NSF (blue, dashed), uncorrected NSF (black, dashed).

In addition to temperature and normal stress profiles, we seek to gain insight into the three integration constants velocity $V_{0}$, heat conduction $q_{0}$ and Knudsen Layer amplitude *A*, depending on the Knudsen number. The three variables are plotted over Kn={0,…,1} in Figure 5.

The signs of velocity $V_{0}$ and heat conduction $q_{0}$ are positive. That is, mass and conductive heat flux are transferred from warm to cold, which means they are transported at $x=-\frac{1}{2}$ into the system via evaporation, and due to the steady state, the same amount of mass and conductive heat is transported at $x=\frac{1}{2}$ out of the system into the colder reservoir via condensation.

The purple, large, dashed line represents R13 with PBC for a=b…=f=1; see Appendix C. Although there are differences in the higher order terms between PBC and MBC, if the adjustable coefficients are set to unity, the order of magnitude of the maximum deviation between the two models is with $\pm 10^{-7}$ very small, i.e., at first glance, both plots appear to be identical.

R13 with PBC shows very good agreement with DSMC for $V_{0}$ and $q_{0}$ for all Knudsen numbers. The PBC results for normal stress are better than those of MBC for Kn<0.3. For higher Knudsen numbers, both PBC and MBC fail to predict σ in precise agreement with DSMC. Again, normal stress cannot be predicted by NSF.

Interestingly, for this PBC fit, Ytrehus’ ratios are similar to those of the MBC, i.e., 1.4% (PBC) and 0.74% (MBC) deviation for $\alpha_{p}$ and 10.02% (PBC) and 10.44% (MBC) for $\alpha_{\theta }$; see Table 3. Corrected NSF is under 1% deviation for both ratios. Uncorrected NSF shows zero deviation for $\alpha_{\theta }$ and 6.18% for $\alpha_{p}$. For Knudsen numbers larger than Kn=0.235, the deviation between DSMC and PBC becomes slightly larger for the temperature profile and stays similar for the normal stress profile. The temperature jump at the boundaries increases with increasing Knudsen number.

### 3.6. Fitting of the Onsager Coefficients: Inverted Temperature Profile

By adjusting the values for ΔT and Δp, it can be shown that the sign of the conductive heat flux $q_{0}$ switches. This leads to an inverted temperature profile as depicted below. The negative sign of $q_{0}$ indicates conductive heat transport from $x=\frac{1}{2}$ to $x=-\frac{1}{2}$; see Figure 1. However, the second law is not violated, since the overall heat transport is given with $Q=\rho V_{0}h+q_{0}$, and the advective term $\rho V_{0}h$ is dominant. Hence, the overall heat *Q* is transported from hot to cold as expected. One notes that due to the reversed sign of the conductive heat flux, the necessary vapourization enthalpy is partly provided by the colder boundary. The liquid temperatures at the boundaries are set to $T_{l}^{0}=1.01$ and $T_{l}^{1}=0.99$ and the respective saturation pressures to $p_{sat}\left(T_{l}^{0}\right)=1.0752$ and $p_{sat}\left(T_{l}^{1}\right)=0.9248$. Therefore, the evaporating material of the system is different from the one considered for the standard temperature profile. The small temperature difference between hot and cold boundaries and the large difference between the saturation pressures allow for a temperature jump large enough to reverse the sign of the conductive heat flux.

By fitting with trial and error, it was not possible to achieve good fits for the standard and inverted temperature profiles at the same time. We believe that this is due to the evaporating material being different between the standard and inverted cases, since the saturation pressures are different. Therefore, we present a fitting for the adjustable factors within the PBC for the inverted case, which is given in Table 4.

The ratios $\alpha_{p}$,$\alpha_{\theta }$, as well as the percentual deviation to Ytrehus’ solution are presented in Table 5.

**Figure 6:**
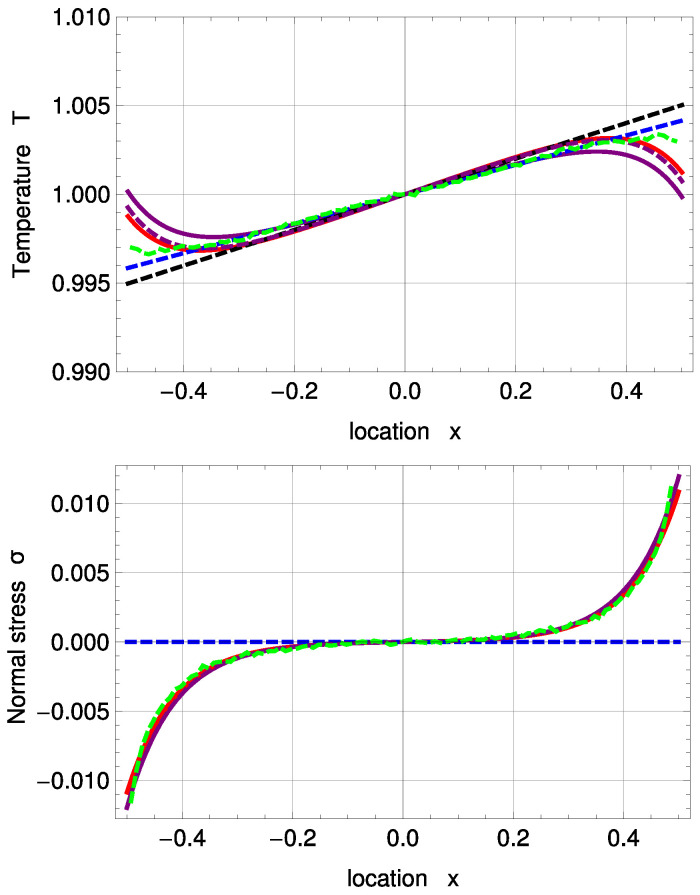
Inverted temperature and normal stress profiles for Kn=0.078 with ΔT=0.01 and Δp=0.075: DSMC (symmetrized; green, dashed), R13 with PBC (purple), R13 with PBC and previous fitting (purple, dashed), R13 with MBC (red), corrected NSF (blue, dashed), uncorrected NSF (black, dashed).

The temperature and stress profiles for Kn=0.078 are given in Figure 6. As a comparison to the new fitting, a PBC solution, which uses the previous coefficients, is given, as well (purple, dashed). R13 with PBC and MBC both overpredict the Knudsen layer at the interfaces. This inaccuracy of Knudsen layer modelling is due to the small number of moments, used in the R13 equations; see [21]. For the temperature profile, corrected NSF shows the best agreement with DSMC here. Normal stress is predicted well for PBC and MBC and is again zero for NSF.

**Figure 7:**
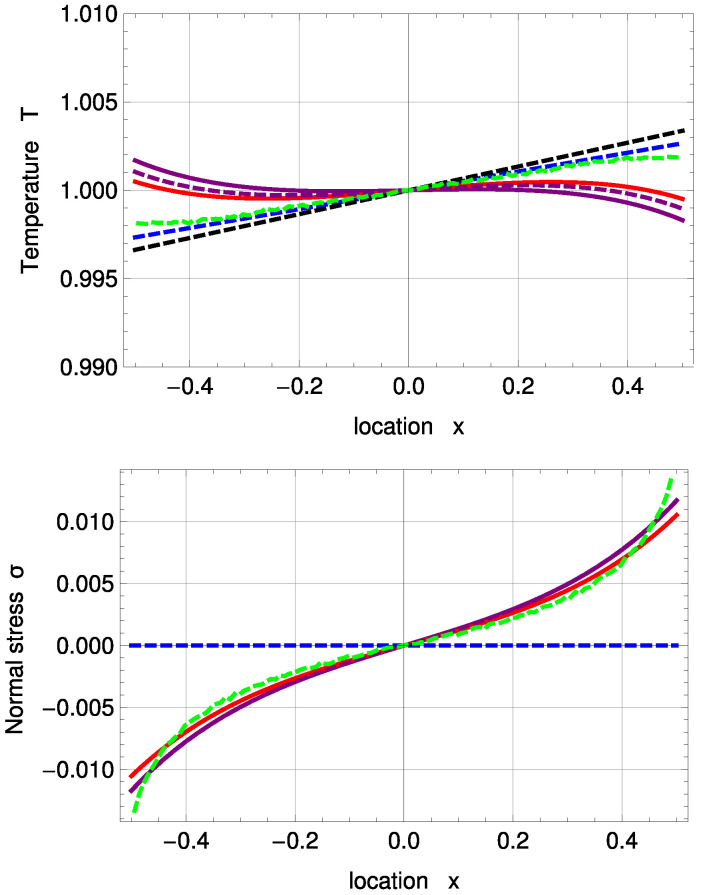
Inverted temperature and normal stress profiles for Kn=0.235 with ΔT=0.01 and Δp=0.075: DSMC (symmetrized; green, dashed), R13 with PBC (purple), R13 with PBC and previous fitting (purple, dashed), R13 with MBC (red), corrected NSF (blue, dashed), uncorrected NSF (black, dashed).

For Kn=0.235, the overprediction of the R13 boundary conditions becomes so large that the profiles are no longer inverted, as shown in Figure 7. Note that it is possible to “turn” the PBC temperature profile to match the DSMC results; however, this leads to worse results for other plots. In this case, MBC shows slightly better results for temperature and normal stress profiles than PBC.

**Figure 8:**
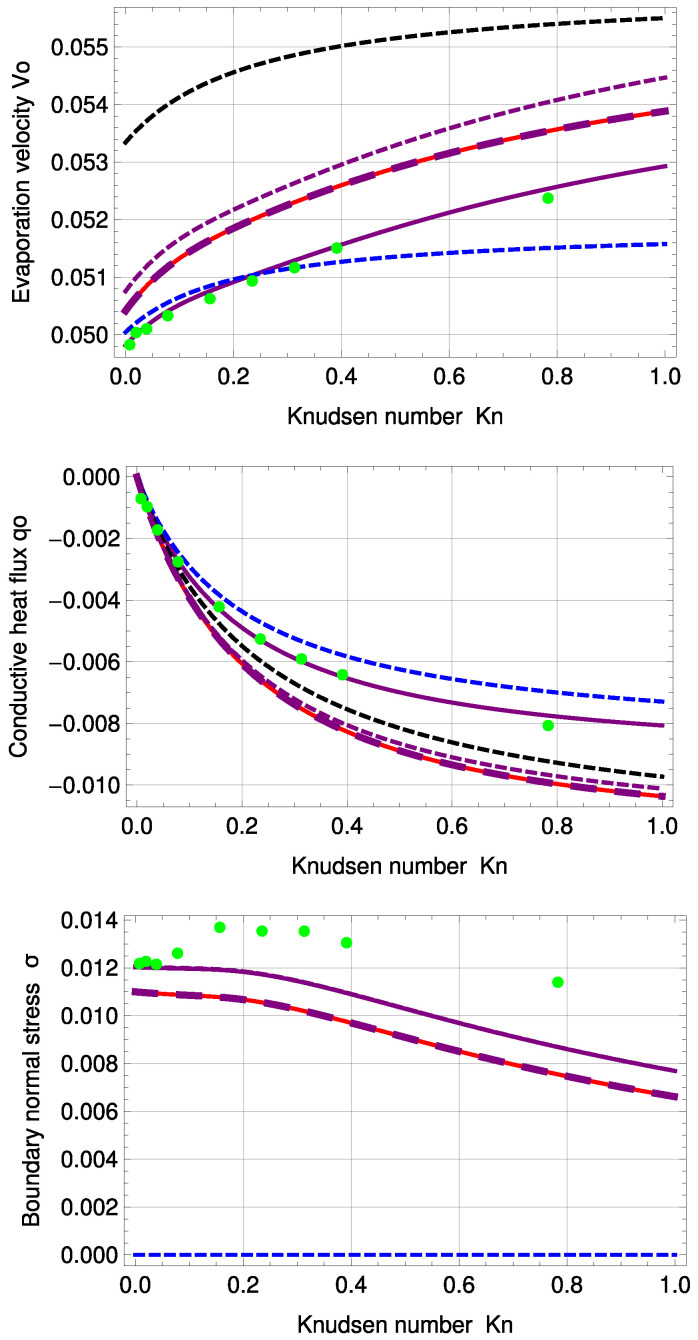
Evaporation velocity $V_{0}$, conductive heat flux $q_{0}$ and boundary normal stress $\sigma_{0}$ for inverted temperature profile: DSMC (green, dots), R13 with PBC (purple), R13 with PBC: a…f=1 (purple, large, dashed), R13 with PBC and previous fitting (purple, dashed), R13 with MBC (red), corrected NSF (blue, dashed), uncorrected NSF (black, dashed). Note: For σ, the purple, dashed line is underneath the purple, solid line.

Figure 8 illustrates velocity, conductive heat flux and normal boundary stress for the inverted temperature profile. The purple, large, dashed line represents R13 with PBC and a=b…=f=1. With an order of magnitude of $\pm 10^{-7}$, in the deviation to the MBC solution, the results of both models are again very similar; see also Figure 5.

For evaporation velocity $V_{0}$ and conductive heat flux $q_{0}$, R13 with PBC is in very good agreement with DSMC. In comparison to the standard temperature profile, the normal boundary stress of the PBC starts to differ from DSMC earlier, i.e., for Kn>0.1. Corrected NSF is in surprisingly good agreement with DSMC for Kn<0.3, but fails to predict normal boundary stress. Except for temperature and normal stress profiles for Kn=0.235, R13 with PBC shows the best agreement with DSMC compared to all discussed models here.

One notes that for this PBC fitting, the deviations of 5.11% in $\alpha_{\theta }$ and 0.46% in $\alpha_{p}$ to Ytrehus’ solution become smaller than for the standard profile.

### 3.7. Impact of Evaporation and Accommodation Coefficients

**Figure 9:**
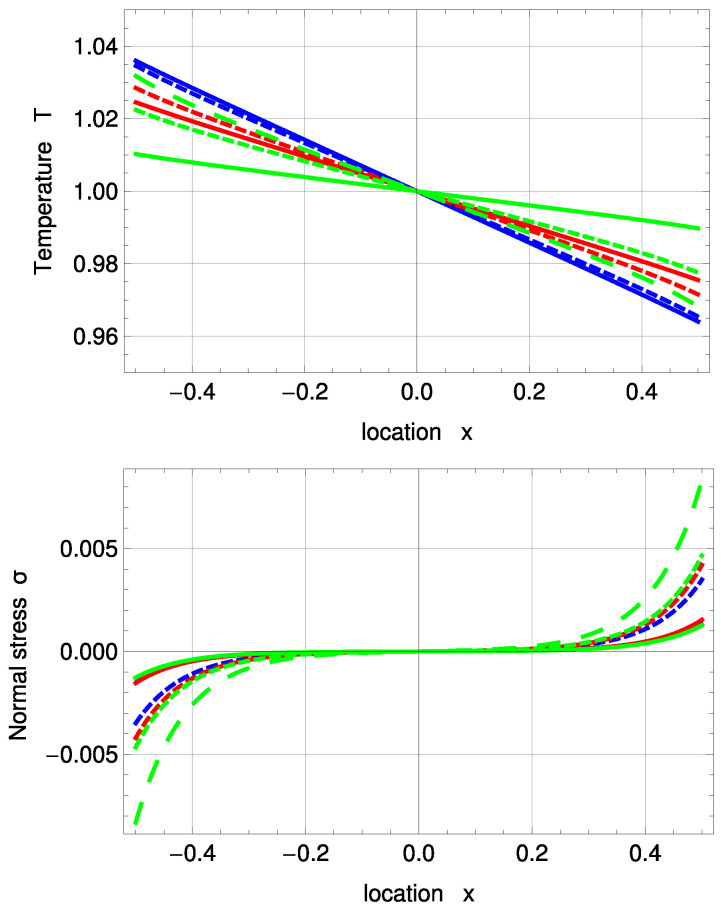
PBC temperature and normal stress profiles for Kn=0.078 and various evaporation and accommodation coefficients: χ=0.1 (green), χ=0.5 (red), χ=1 (blue), ϑ=0.1 (solid), ϑ=0.5 (dashed), ϑ=1 (large, dashed). Note: For ϑ=1, the green, large dashed curve represents the solutions of all three χ.

To gain a better understanding of the impact of evaporation and accommodation coefficients, the PBC shall be tested for the standard temperature profile of the previously discussed problem and a variety of ϑ, χ. Figure 9 illustrates solutions of the PBC for Problem I (Section 3.3) together with the fitting from Table 2 and Kn=0.078. The plots are based on χ=0.1 (green), χ=0.5 (red), χ=1 (blue), ϑ=0.1 (solid), ϑ=0.5 (dashed) and ϑ=1 (large dashed).

For ϑ=1, the solutions are independent of χ. Since the evaporation coefficient is defined through the condensation coefficient, this may be explained due to the fact that for the condensation coefficient being unity, no reflection occurs, and all vapour molecules hitting the liquid interface are condensed. The largest temperature jump between gas and the boundary is found for ϑ=0.1 and χ=0.1 and the smallest for χ=1.

**Figure 10:**
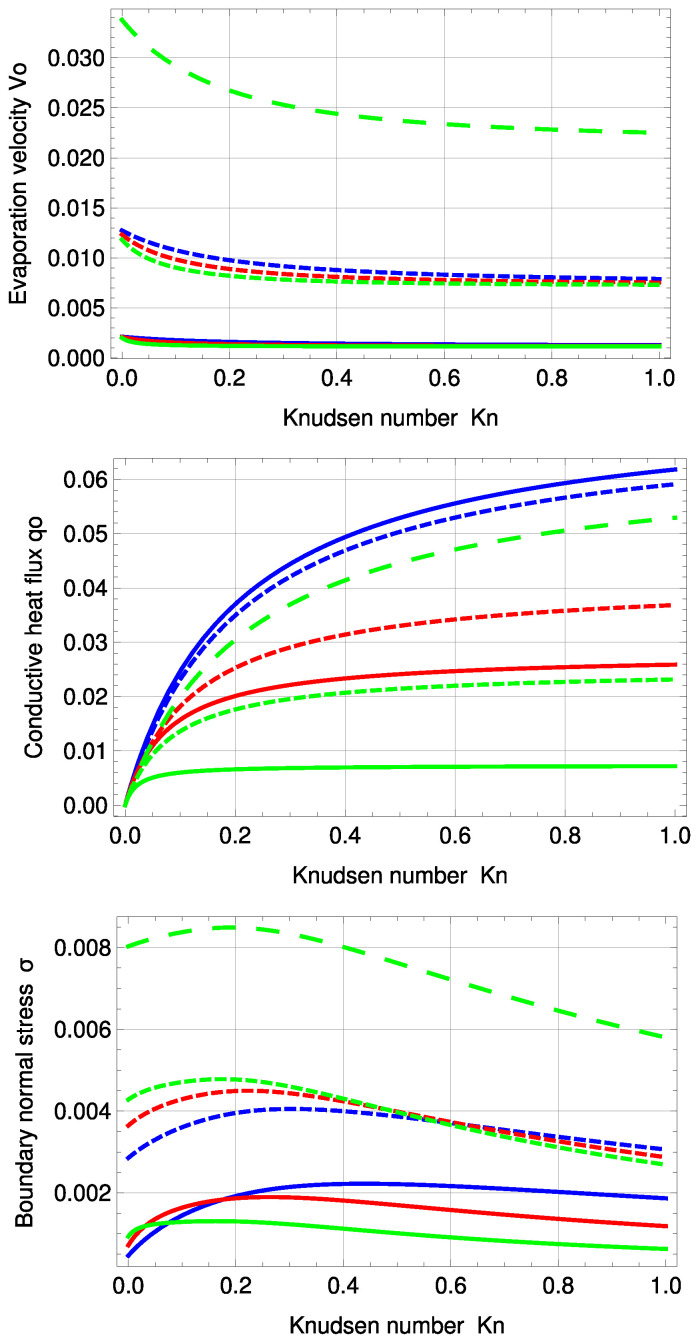
PBC evaporation velocity $V_{0}$, conductive heat flux $q_{0}$ and boundary normal stress $\sigma_{0}$ for the standard temperature profile and various evaporation and accommodation coefficients: χ=0.1 (green), χ=0.5 (red), χ=1 (blue), ϑ=0.1 (solid), ϑ=0.5 (dashed), ϑ=1 (large, dashed). Note: For ϑ=1, the green, large dashed curve represents the solutions of all three χ.

The stress profile seems to be dependent mainly on the evaporation coefficient. The accommodation coefficient has only a small impact for ϑ=0.5. The largest stress can be found for ϑ=1. Evaporation velocity $V_{0}$, conductive heat flux $q_{0}$ and boundary normal stress σ for various values of ϑ and χ are depicted in Figure 10.

The results of $V_{0}$ seem to be almost independent of χ, except for ϑ=0.5, where χ has a small impact. Interestingly, χ has a large influence on $q_{0}$ and σ, particularly for ϑ=0.1.

### 3.8. Notes on the Meaning of the Individual Onsager Coefficients of the Normal Fluxes

The fittings used in the Table 2 and Table 4 are based on a trial and error procedure, in which the factors a…f within the Onsager coefficients (31)–(36) are individually adjusted. Due to symmetry of the Onsager matrix, six independent parameters need to be determined. The tuning of the Onsager coefficients one by one gives an insight into their respective impact. However, one notes that due to the coupling within the Onsager matrix in Equation (28), the individual Onsager coefficient impacts multiple fluxes. The following is an attempt to highlight some trends, which were observed during the fitting procedure.

Since $\lambda_{0}$ appears only in the equation for the normal velocity, it has a strong impact on $V_{0}$ and no impact on the conductive heat flux $q_{0}$. Apparently, it has no impact on the boundary normal stress σ. Temperature and stress profiles appear to be independent of $\lambda_{0}$ as well. The coefficient $\lambda_{1}$ has a big impact on $V_{0}$ and $q_{0}$ and a small impact on σ. It has a major impact on the temperature profile and a smaller impact on the stress profile. $\lambda_{2}$ strongly influences $V_{0}$ and σ and very slightly $q_{0}$. Since $\lambda_{2}$ does not appear in the equation for $q_{0}$, this is expected. It has an impact on temperature and stress profiles, but with clear emphasis on the stress profile.

The coefficient $\lambda_{3}$ seems to play a key role in the fitting. Even though it appears only in the equation for $q_{0}$, it has not only a strong impact on the magnitude and slope of $q_{0}$, but also on those of $V_{0}$ and σ. Regarding the profiles, $\lambda_{3}$ seems to impact mainly the temperature and only very slightly the stress. The Onsager coefficient $\lambda_{4}$ mainly impacts σ, but also $V_{0}$, $q_{0}$ and both profiles, with stronger impact on the stress profile, as expected. $\lambda_{5}$ appears only in the equation for the normal component of the higher moment $m_{nnn}$. The coefficient has a strong impact on σ, a medium impact on $V_{0}$ and no impact on $q_{0}$. It influences the stress profile significantly and the temperature profile slightly.

After these dependencies were established, several rounds of fitting were done, until a reasonable fitting was obtained.

## 4. Evaporation in Numerical Two-Dimensional Steady-State Simulation

### 4.1. R13 with Onsager Boundary Conditions in Numerical Simulation

It shall be shown that the applicability of R13 with PBC (Phenomenological Boundary Conditions) is not limited to one-dimensional systems. The code of Torrilhon and Sarna [22], written in C++, is used in this section to solve the R13 equations with PBC for evaporation. As a comparison, simplified NSF (Navier–Stokes–Fourier) is solved with the same program. Torrilhon and Sarna’s code allows for generic implementation of macroscopic transport equations. The numerical solver relies on a Discontinuous Galerkin (DG) method, which utilizes finite elements to discretize the system. Here, the code is extended by implementing the evaporation boundary conditions previously derived in Section 3 and also simplified Onsager boundary conditions for NSF.

The PBC for R13, given in Equations (28)–(30), are adjusted by using data for Maxwell molecules out of Table 1. The liquid phase is not solved and therefore can be treated in the same manner as a wall, which allows for mass transfer. Adjustment of the Onsager coefficients allows one to derive other boundary conditions, such as the wall with energy transfer or inflow/outflow. Table 6 gives an overview of these modifications.

For an adiabatic wall (fully specular reflective), all Onsager coefficients are set to zero, which leads to $v_{n}^{g}=q_{n}^{g}=m_{nnn}={\bar{\sigma }}_{nk}^{g}={\bar{R}}_{nk}={\tilde{m}}_{nij}=0$. The Onsager coefficients for a wall with energy transfer are taken from [12]. The adjustable coefficients within the Onsager coefficients for the different boundaries were already implemented in Table 6.

Note: Compared to Section 3.1, a slightly different fitting is used here. Additionally, the coefficients used in $\lambda_{0},\ldots ,\lambda_{5}$ are based on adjustments as in Problem I (Section 3.3); however, different definitions of the Knudsen number between DSMC and R13 were used. Therefore, a small error is introduced here.

The coefficients in $\zeta_{0},\ldots ,\zeta_{2}$ and $\kappa_{0}$ are not fitted and set to unity. The adjustable coefficients for a wall with energy transfer $\lambda_{3},\ldots ,\lambda_{5}$ and $\zeta_{0},\ldots ,\zeta_{2}$ are taken from [12], and $\kappa_{0}$ is set to unity here. Depending on the boundary, different pressures and temperatures are assumed, as depicted in Table 7.

For a detailed description of the numerical solution, see [22].

### 4.2. Navier–Stokes–Fourier with Onsager Boundary Conditions in Numerical Simulation

For obtaining a comparison to the R13 solutions for two-dimensional systems, the Navier–Stokes–Fourier equations together with Onsager boundary conditions for evaporation/condensation are used here. For χ=ϑ=1 and considering one-dimensional geometry, evaporation boundary conditions for NSF are given in Appendix D; see (A29). For two- and three-dimensional geometries, an additional boundary condition is found in [11] and reads:

(66) $${\bar{\sigma }}_{nk}^{g}=-\frac{\vartheta +\chi (1-\vartheta )}{2-\vartheta -\chi (1-\vartheta )}\sqrt{\frac{2}{\pi RT}}\left(p{\bar{v}}_{k}^{g}+\frac{1}{5}{\bar{q}}_{k}^{g}\right).$$

Note that Equations (A29) are simplified equations for 1D geometry. Again, by considering χ=ϑ=1 and after full linearization and non-dimensionalization, Equation (66) becomes:

(67) $${\bar{\sigma }}_{nk}^{g}=-\sqrt{\frac{2}{\pi }}\left({\bar{v}}_{k}^{g}+\frac{1}{5}{\bar{q}}_{k}^{g}\right).$$

### 4.3. Numerical Solutions for Two-Dimensional Channel-Flow with four Evaporating Cylinders

**Figure 11:**
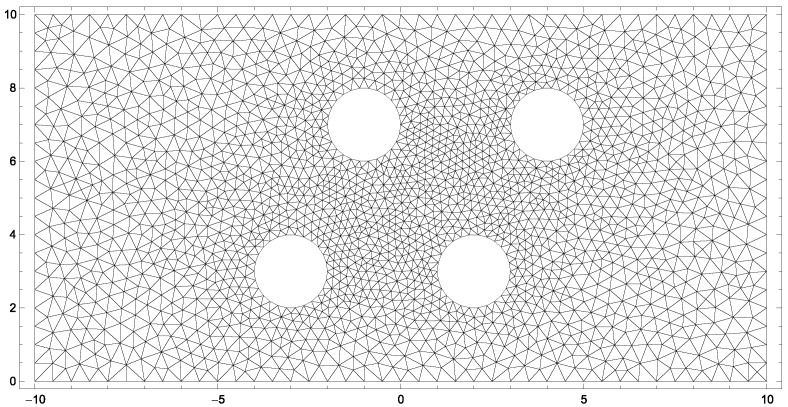
Grid of two-dimensional channel-flow with four evaporating cylinders.

The system of interest for the two-dimensional, steady-state simulation is a channel with four evaporating cylinders, which is discretized as depicted in Figure 11.

The left boundary is the inlet of the channel flow, and the right boundary is the outlet. The top and bottom are walls, which allow energy transfer. The cylinder walls use evaporation boundary conditions given by (28)–(30) with Table 6 for R13 and (67), (A29) and (A31) for NSF.

The input parameters, which are given in Table 8, are non-dimensional and describe the deviation to equilibrium. They are chosen in a way that evaporation at the cylinders can be observed clearly.

**Figure 12:**
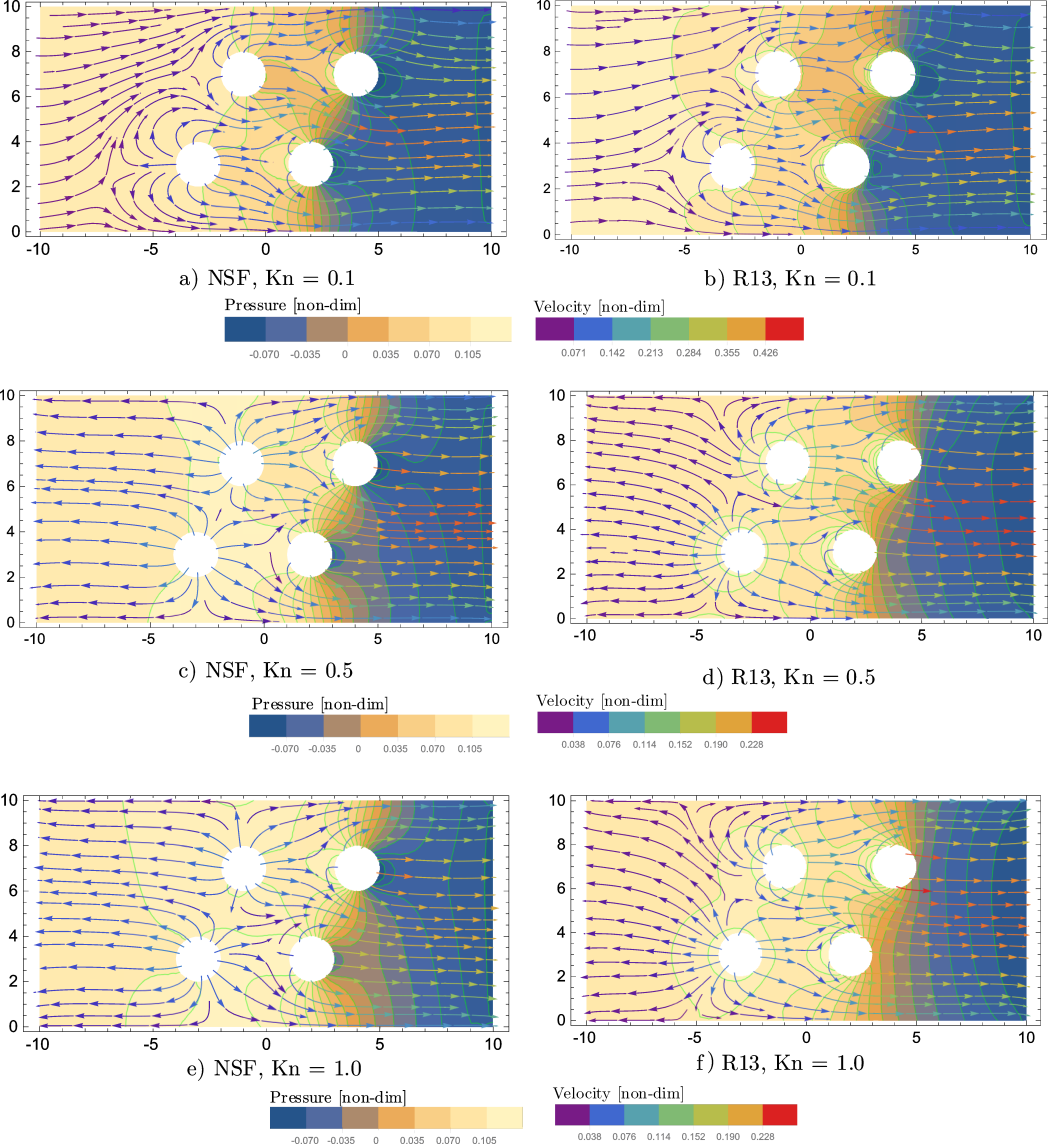
Pressure contours superimposed by velocity streamlines for two-dimensional channel-flow with four evaporating cylinders and various Knudsen numbers.

The plots in Figure 12 show pressure contours, superimposed by velocity streamlines, for R13 and NSF, for the three Knudsen numbers: Kn={0.1, 0.5, 1}.

For Kn=0.1, the velocity streamlines are similar between R13 and NSF. The inflow of the left boundary collides with the evaporating flow, which leaves the two cylinders on the left-hand side. The largest flow velocity is observed in between the two cylinders on the right-hand side. For Kn=0.5, the evaporation overcomes the inflow and leaves the system at the inlet of the channel. This interesting effect is observed for R13 and NSF, but with different flow behaviour. For R13, the streamlines, which leave the inlet, have their origin mainly in the left bottom cylinder. The dominance of the left cylinder of R13 becomes even more apparent for Kn=1. The NSF velocity streamlines at the inlet for Kn={0.5,1} come almost equally from both cylinders on the left-hand side.

For Kn=0.1, the pressure contours of R13 and NSF show very similar behaviour. With increasing Kn, the R13-pressure contours on the right-hand side of the diagrams disconnect from each other and become almost vertical for Kn=1.

**Figure 13:**
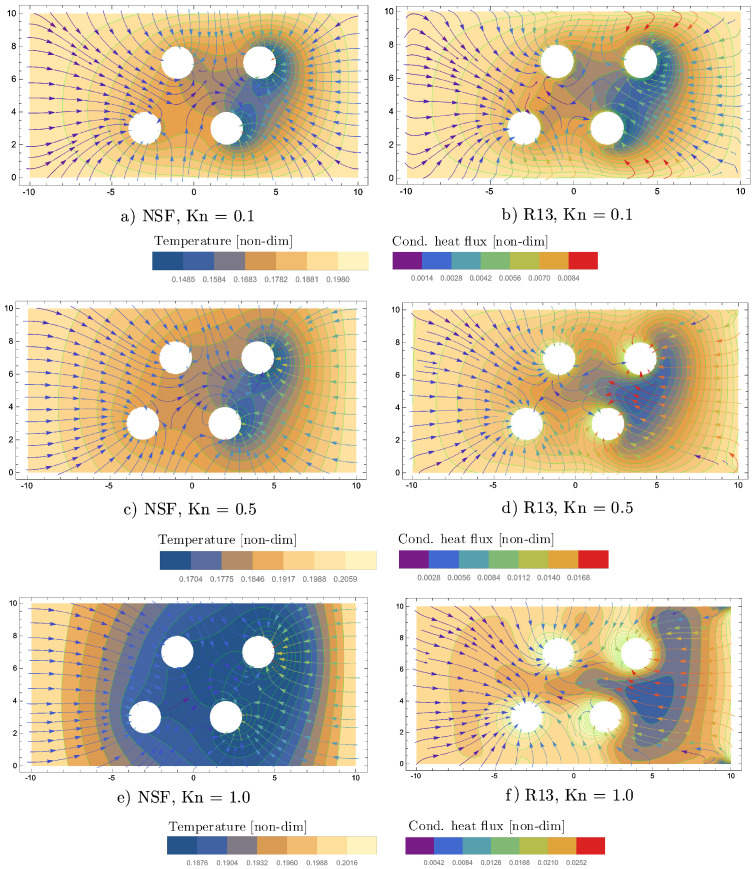
Temperature contours superimposed by cond.heat flux streamlines for two-dimensional channel-flow with four evaporating cylinders and various Knudsen numbers.

Furthermore, for Kn=1, significant differences between R13 and NSF are found for the temperature profiles, which are depicted in Figure 13.

The overall temperature around the four evaporating cylinders is much lower for NSF than for R13. As can be seen by the conductive heat flux streamlines, the enthalpy of vapourization is provided by the boundaries, as in the previous simulations. The magnitude of the R13 heat flux shows interesting peaks in between the two cylinders on the right-hand side for Kn={0.5,1}.

The large differences between R13 and NSF for Kn={0.5,1} are likely due to rarefaction effects, which cannot be captured by NSF. It has to be taken into account, as mentioned in Section 4.2, that simplified NSF boundary conditions are used here. Note that R13 is limited to flow regimes below Kn=1 and can only describe a tendency here. For validation of the R13 results, a reliable reference, such as from a DSMC simulation, is necessary, which might be part of future work.

## 5. Conclusions

Based on the Onsager theory, which utilizes the second law of thermodynamics, evaporation boundary conditions (PBC) for the R13 equations are derived. The Onsager coefficients have been determined by following a process consisting of three steps: In the first step (Section 3.1), the boundary conditions are compared with previously discussed boundary conditions for evaporation (MBC), which represent an alternative approach for deriving boundary conditions for R13. Under the assumption of proper results for MBC in the Navier–Stokes–Fourier (NSF) regime and by keeping in mind that higher moments develop a significant impact only for higher Knudsen numbers, coefficients are taken over from MBC to PBC so that the differences between the sets of boundary conditions lie only in the terms with higher moments [12]. The idea is to find boundary conditions that are just as reliable as MBC in the NSF regime and more accurate in the rarefied gas regime. In the next step, adjustable coefficients are suggested for the PBC. These coefficients are fitted by trial and error to DSMC data for the analytical solution of a finite, one-dimensional system (Section 3.3). In the third step for finding meaningful Onsager coefficients, the half space problem (Section 3.4) is solved analytically, and ratios suggested by Ytrehus [15] are used to fine-tune the coefficients. The overall agreement between PBC and DSMC (Section 3.5 and Section 3.6) has been shown to be better than for MBC/NSF and DSMC. Even though there are differences in the higher order terms, when setting the adjustable coefficients a…f of the PBC to unity, the maximum deviation to the MBC, for the boundary values of the finite problem, is in the order of magnitude of $\pm 10^{-7}$, only.

For a general approach to convert MBC to PBC, with differences in the higher order terms only, see [17]. Kinetic boundary conditions, such are used in [6,11,21,23,24], might lead to violation of the Onsager symmetry relations. Furthermore, due to the approximative nature of the models, there can be small inaccuracies in the results, e.g., due to the details of the Knudsen layers that cannot be fully described [21]. The present approach uses fitting of coefficients to recover Onsager symmetry and also to improve the accuracy of the results by small adjustments of the kinetic coefficients.

The impact of the evaporation and accommodation coefficients is discussed in Section 3.7. In Section 3.8, it is explained how the trial and error fitting gives an insight into the meaning of the individual Onsager coefficients.

Due to lack of a mathematical approach for the fitting, i.e., an optimization algorithm, it is uncertain if significantly better fittings for the presented problems are possible. This may be part of a future analysis. Even though NSF fails to predict normal stress for the presented systems, it shows surprisingly good results for low to moderate Knudsen numbers. The advantage of R13 with PBC compared to NSF might be shown even more clearly in numerical simulations for complex geometries. The Onsager coefficients appear to be dependent on the evaporating material, which in the practical application becomes problematic. Therefore, we recommend an investigation considering the fitting of Onsager coefficients as a function of the enthalpy of vapourization, which defines the material.

In Section 4, the new evaporation/condensation boundary conditions are implemented in a code for the numerical solution of two-dimensional, steady-state problems. Results for Knudsen numbers of Kn = {0.1, 0.5, 1.0} are obtained and compared to simplified Navier–Stokes–Fourier solutions. It is observed that with increasing Knudsen number, R13 shows different flow behaviour than NSF.

It is necessary to compare these results to a reliable reference, such as a DSMC solution, which shall be a future effort. Additionally, it might be of interest to compare the numerical R13 results to those of a 26-moment method; see [25].

## Figures and Tables

**Table 1:** Coefficients for Maxwell (MM), Hard Sphere (HS) and Bhatnagar–Gross–Krook (BGK) models for the R13 equations.

	$\varpi_{2}$	$\varpi_{3}=\theta_{4}$	$\theta_{2}$	Pr	Pr${}_{R}$	Pr${}_{M}$	Pr${}_{\Delta }$
MM	2	3	45/8	2/3	7/6	3/2	2/3
BGK	2	2	5/2	1	1	1	1
HS	2.02774	2.42113	5.81945	0.6609	1.3307	1.3951	0.9025

**Table 2:** Factors to adjust the Onsager coefficients of the Phenomenological Boundary Conditions (PBC) for the standard temperature profile.

	a	b	c	d	e	f
PBC standard profile	1.02	0.96	1.30	0.94	0.50	1.20

**Table 3:** Solutions for Ytrehus’ ratios and percentual deviation to Ytrehus’ solution for the standard temperature profile. MBC, Macroscopic Boundary Conditions; NSF, Navier–Stokes–Fourier.

	$\alpha_{p}$	% to Ytrehus	$\alpha_{\theta }$	% to Ytrehus
PBC standard profile	2.0956	1.40	0.4875	10.02
MBC	2.1097	0.74	0.4894	10.44
NSF	1.9940	6.18	0.4431	-
NSF corrected	2.1254	-	0.4472	0.93
Ytrehus	2.1254	-	0.4431	-

**Table 4:** Factors to adjust the Onsager coefficients of the PBC for the inverted profile.

	a	b	c	d	e	f
PBC inverted profile	0.983	0.83	1.30	0.87	0.50	1.20

**Table 5:** Solutions for Ytrehus’ ratios and percentual deviation to Ytrehus’ solution for inverted profile.

	$\alpha_{p}$	% to Ytrehus	$\alpha_{\theta }$	% to Ytrehus
PBC inverted profile	2.1352	0.46	0.4657	5.11
Ytrehus	2.1254	-	0.44311	-

**Table 6:** Derivation of boundary conditions by adjusting the Onsager coefficients.

	E Vapouration/Condensation	W All with Energy Transfer	I Inflow/Outflow
$\lambda_{0}$	$0.975\vartheta_{2}$	0	$1/10^{-5}$
$\lambda_{1}$	$-0.4375\vartheta_{2}$	0	0
$\lambda_{2}$	$-0.4\vartheta_{2}$	0	0
$\lambda_{3}$	$2.2\chi_{2}$	$1.744\vartheta_{2}$	$1/10^{-5}$
$\lambda_{4}$	$-0.28\chi_{2}$	$-1.744\vartheta_{2}$	0
$\lambda_{5}$	$2.184\chi_{2}+0.28\vartheta_{2}$	$2\vartheta_{2}$	0
$\zeta_{0}$	$\chi_{2}\;(\mathrm{Not}\;\mathrm{fitted})$	$0.9143\vartheta_{2}$	1.0(Notfitted)
$\zeta_{1}$	$-\chi_{2}\;(\mathrm{Not}\;\mathrm{fitted})$	$-0.9143\vartheta_{2}$	1.0(Notfitted)
$\zeta_{2}$	$13\chi_{2}\;(\mathrm{Not}\;\mathrm{fitted})$	$\vartheta_{2}$	1.0(Notfitted)
$\kappa_{0}$	$2\chi_{2}\;(\mathrm{Not}\;\mathrm{fitted})$	$2\vartheta_{2}\;(\mathrm{Not}\;\mathrm{fitted})$	1.0(Notfitted)

**Table 7:** Overview of input parameters for the boundary conditions.

	E Vapouration/Condensation	W All with Energy Transfer	I Inflow/Outflow
$p_{sat}$	$p_{evap}$	−	$\pm p_{flow}$
$T_{l}$	$T_{evap}$	$T_{w}$	$T_{flow}$

**Table 8:** Input parameters for two-dimensional channel flow with four evaporating cylinders.

	E Vapouration/Condensation	W All with Energy Transfer	I Inflow/Outflow
$p_{sat}$	$p_{evap}=0.2$	−	$\pm p_{flow}=0.1$
$T_{l}$	$T_{evap}=0.2$	$T_{w}=0.2$	$T_{flow}=0.2$

## Appendix A. Normal and Tangential Components

Within the process of deriving Onsager boundary conditions, it is desirable to decompose the tensors into their respective normal and tangential components. The normal component of a vector can be defined as:

(A1) $$q_{n}=q_{k}n_{k},$$

with its tangential component:

(A2) $${\bar{q}}_{i}=q_{i}-q_{n}n_{i},\;\mathrm{with}\;{\bar{q}}_{i}n_{i}=0\;.$$

Similarly, one may define the components of a symmetric and trace-free tensor as [12]:

(A3) $$\sigma_{nn}=\sigma_{rk}n_{k}n_{r},$$

(A4) $${\bar{\sigma }}_{ni}=\sigma_{ik}n_{k}-\sigma_{nn}n_{i},\;\mathrm{with}\;{\bar{\sigma }}_{ni}n_{i}=0,$$

(A5) $${\tilde{\sigma }}_{ij}=\sigma_{ij}-\sigma_{nn}\left(\frac{3}{2}n_{i}n_{j}-\frac{1}{2}\delta_{ij}\right)-{\bar{\sigma }}_{ni}n_{j}-{\tilde{\sigma }}_{nj}n_{i},\;\mathrm{with}\;{\tilde{\sigma }}_{ij}n_{j}={\tilde{\sigma }}_{kk}=0.$$

Here, $\sigma_{nn}$ is the normal-normal component, ${\bar{\sigma }}_{ni}$ the normal-tangential component and ${\tilde{\sigma }}_{ij}$ the tangential-tangential component. As mentioned in Section 1.2, the Einstein notation does not apply for index *n*. Similarly for a symmetric and trace-free third order tensor, i.e., a three-dimensional matrix, one finds:

(A6) $$m_{nnn}=m_{ijk}n_{i}n_{j}n_{k},$$

(A7) $${\bar{m}}_{nni}=m_{ijk}n_{j}n_{k}-m_{nnn}n_{i},\;\mathrm{with}\;{\bar{m}}_{nni}n_{i}=0,$$

(A8) $${\tilde{m}}_{nij}=m_{ijk}n_{k}-m_{nnn}\left(\frac{3}{2}n_{i}n_{j}-\frac{1}{2}\delta_{ij}\right)-{\bar{m}}_{nni}n_{j}-{\bar{m}}_{nnj}n_{i},\;\mathrm{with}\;{\tilde{m}}_{nij}n_{j}=0.$$

Additionally, one has:

(A9) $$\delta_{ij}{\bar{m}}_{nnj}n_{i}=\delta_{ij}{\bar{\sigma }}_{nj}n_{i}=\delta_{ij}{\tilde{m}}_{nij}=0,$$

(A10) $$\delta_{ij}n_{i}n_{j}=n_{j}n_{j}=1.$$

## Appendix B. Derivation of Entropy Fluxes

Based on the incompressible Navier–Stokes–Fourier equations, a reduced entropy flux $\Psi_{k}^{l}$ for the liquid side of a liquid-gas interface shall be derived in the following. Here, the vapour is a monatomic ideal gas with specific heat $c_{p}=\frac{5}{2}R$, and the liquid is described as an incompressible simple liquid. The heat of vapourization at reference state $T_{0}$, $p_{sat}\left(T_{0}\right)$ is:

(A11) $$h_{gl}^{0}=h^{g}\left(T_{0}\right)-h^{l}\left(T_{0}\right)=\frac{5}{2}RT_{0}-\left(c_{l}T_{0}+\frac{p_{sat}\left(T_{0}\right)}{\rho_{l}}+h_{0}\right),$$

with the enthalpies:

(A12) $$h^{l}=c_{l}\left(T-T_{0}\right)+\frac{5}{2}RT_{0}+\frac{p-p_{sat}\left(T_{0}\right)}{\rho_{l}}-h_{gl}^{0},$$

(A13) $$h^{g}=\frac{5}{2}RT.$$

The energy density of the liquid $\varepsilon^{l}=\rho_{l}u^{l}$, with $u^{l}$ as the internal energy, is:

(A14) $$\varepsilon^{l}=\rho_{l}\left(h^{l}-\frac{p}{\rho_{l}}\right)=\rho_{l}\left(c_{l}\left(T-T_{0}\right)+\frac{5}{2}RT_{0}-\frac{p_{sat}\left(T_{0}\right)}{\rho_{l}}-h_{gl}^{0}\right).$$

The entropy density $\eta^{l}=\rho_{l}s^{l}$ of the incompressible liquid is given as:

(A15) $$\eta^{l}=c_{l}\rho_{l}\ln\frac{T^{l}}{T_{0}}-\frac{\rho_{l}}{T_{0}}h_{gl}^{0},$$

where the proper entropy difference at equilibrium state $\frac{\eta^{v}\left(T_{0}\right)}{\rho^{v}}-\frac{\eta^{l}\left(T_{0}\right)}{\rho_{l}}=\frac{h_{gl}^{0}}{T_{0}}$ was used. The conservation laws for mass, energy and entropy for a fluid are:

(A16) $$\frac{\partial \rho }{\partial t}+\frac{\partial \rho v_{k}}{\partial x_{k}}=0,$$

(A17) $$\frac{\partial \left(\varepsilon +\frac{\rho }{2}v^{2}\right)}{\partial t}+\frac{\partial \left(\left(\varepsilon +\frac{\rho }{2}v^{2}\right)v_{k}+q_{k}+pv_{k}+\sigma_{ik}v_{i}\right)}{\partial x_{k}}=0,$$

(A18) $$\frac{\partial \eta }{\partial t}+\frac{\partial \left(\eta v_{k}+\phi_{k}\right)}{\partial x_{k}}=\sigma_{gen},$$

with $\eta v_{k}+\phi_{k}=\Psi_{k}$ as the sum of convective and conductive entropy flux. When one intends linearized balance laws, the entropy must be considered up to quadratic terms in deviations from equilibrium. Motivated by entropy for the vapour given in [19], η is replaced by a linear combination α:

(A19) $$\alpha =\eta +\frac{5}{2}R\rho -\frac{1}{T_{0}}\left(\varepsilon +\frac{\rho }{2}v^{2}\right),$$

which obeys the balance laws (A16)–(A18). Then, the reduced entropy balance reads:

(A20) $$\frac{\partial \alpha }{\partial t}+\frac{\partial \left(\alpha v_{k}+\phi_{k}-\frac{1}{T_{0}}\left(pv_{k}+q_{k}+\sigma_{ik}v_{i}\right)\right)}{\partial x_{k}}=\Sigma_{gen}.$$

For deriving the entropy flux on the liquid side, incompressible NSF is used with $\phi_{k}=\frac{q_{k}^{l}}{T^{l}}$ for the conductive part of the entropy flux. Hence, the reduced entropy flux can be read from (A20) as:

(A21) $$\Omega_{k}^{l}=\alpha^{l}v_{k}^{l}+\frac{q_{k}^{l}}{T^{l}}-\frac{1}{T_{0}}\left(q_{k}^{l}+p^{l}v_{k}^{l}+\sigma_{ik}^{l}v_{i}^{l}\right).$$

By using the equations of state for a liquid, (A14) and (A15) in (A19) and after linearizing and non-dimensionalizing with (1), the reduced entropy density ${\tilde{\eta }}^{l}$ obtains the form:

(A22) $${\tilde{\eta }}^{l}=\frac{\alpha^{l}}{R\rho_{l}}=\frac{p_{sat}\left(T_{0}\right)}{\rho_{l}RT_{0}}-\frac{c_{l}}{R}\frac{{\left({\hat{T}}^{l}\right)}^{2}}{2}-\frac{1}{2}{\left({\hat{v}}^{l}\right)}^{2}.$$

The reduced entropy flux (dimensionless, linearized) on the liquid side, which, depending on evaporation or condensation, either enters or leaves the interface between liquid and vapour, follows as:

(A23) $$\Psi_{k}^{l}=\frac{\Omega_{k}^{l}}{\rho_{0}R\sqrt{RT_{0}}}=-{\hat{p}}^{l}{\hat{v}}_{k}^{l}-{\hat{q}}_{k}^{l}{\hat{T}}^{l}-{\hat{\sigma }}_{ik}^{l}{\hat{v}}_{i}^{l}.$$

The hats, which denote dimensionless deviations from the respective equilibrium state, are neglected in Section 3. By considering R13 for the vapour phase, the entropy for vapour can be found in the same manner, over a linear combination of (A16)–(A18). However, due to the higher moments, there are additional terms in the (dimensionless, linearized) reduced entropy density ${\tilde{\eta }}^{g}$ and reduced entropy flux $\Psi_{k}^{g}$; see [19]:

(A24) $${\tilde{\eta }}^{g}={\hat{\eta }}_{0}-\frac{{\left({\hat{\rho }}^{g}\right)}^{2}}{2}-\frac{{\left({\hat{v}}^{g}\right)}^{2}}{2}-\frac{3}{4}{\left({\hat{T}}^{g}\right)}^{2}-\frac{\varpi_{2}}{8}{\left({\hat{\sigma }}^{g}\right)}^{2}-\frac{2\theta_{2}}{25}{\left(\mathrm{Pr}\right)}^{2}{\left({\hat{q}}^{g}\right)}^{2},$$

(A25) $$\Psi_{k}^{g}=-{\hat{p}}^{g}{\hat{v}}_{k}^{g}-{\hat{q}}_{k}^{g}{\hat{T}}^{g}-{\hat{\sigma }}_{ik}^{g}{\hat{v}}_{i}^{g}-\frac{\varpi_{3}}{5}\mathrm{Pr}{\hat{q}}_{i}^{g}{\hat{\sigma }}_{ik}^{g}-\frac{\varpi_{2}}{4}{\hat{\sigma }}_{ij}^{g}{\hat{m}}_{ijk}-\frac{2\theta_{2}}{25}{\left(\mathrm{Pr}\right)}^{2}\left({\hat{q}}_{i}^{g}{\hat{R}}_{ik}+\frac{\hat{\Delta }}{3}{\hat{q}}_{k}^{g}\right).$$

## Appendix C. Comparison PBC vs. MBC for Non-Fitted Coefficients

For Maxwell molecules, the normal boundary conditions of PBC and MBC are compared with each other. The Onsager coefficients (31)–(36) are plugged into the PBC, which consist of normal components (28), while considering data for Maxwell molecules from Table 1 and setting the adjustable coefficients a=b=…=f=1:

(A26) $$V_{n}^{g}=\sqrt{\frac{2}{\pi }}\frac{\vartheta }{2-\vartheta }\left(p_{sat}\left(T^{l}\right)-p^{g}-\frac{1}{2}\sigma_{nn}^{g}+\frac{1}{2}\left(T^{g}-T^{l}\right)+\underline{\frac{1}{30}\Delta }+\underline{\frac{1}{10}R_{nn}}\right),$$

(A27) $$q_{n}^{g}=-\sqrt{\frac{2}{\pi }}\frac{\vartheta +\chi (1-\vartheta )}{2-\vartheta -\chi (1-\vartheta )}\left(2\left(T^{g}-T^{l}\right)+\frac{1}{2}\sigma_{nn}^{g}+\underline{\frac{2}{15}\Delta }+\underline{\frac{2}{5}R_{nn}}\right)-\frac{1}{2}V_{n}^{g},$$

(A28) $$m_{nnn}=\sqrt{\frac{2}{\pi }}\frac{\vartheta +\chi (1-\vartheta )}{2-\vartheta -\chi (1-\vartheta )}\left(\frac{2}{5}\left(T^{g}-T^{l}\right)-\frac{7}{5}\;\sigma_{nn}^{g}+\underline{\frac{2}{75}\Delta }+\underline{\frac{2}{25}R_{nn}}\right)-\frac{2}{5}V_{n}^{g}.$$

The terms that are different between PBC and MBC are underlined. All lower order terms, i.e., $p^{g}$, $\sigma_{nn}$ and $\left(T^{g}-T^{l}\right)$, are equal between PBC and MBC, whereas the higher order terms Δ and $R_{nn}$ differ; see Section 1.2.

## Appendix D. Onsager Boundary Conditions for Navier–Stokes–Fourier

Here, the Navier–Stokes–Fourier equations are used together with evaporation boundary conditions, based on the Onsager theory. For full evaporation ϑ=1, fully-diffusive reflection χ=1 and by considering one-dimensional heat and mass transfer only, the boundary conditions are given as [11,26]:

(A29) $$\left[\begin{array}{c} \frac{p_{sat}-p^{g}}{\sqrt{2\pi }}\\ \frac{\left(T^{l}-T^{g}\right)}{\sqrt{2\pi }} \end{array}\right]=\left[\begin{array}{cc} r_{11} & r_{12}\\ r_{21} & r_{22} \end{array}\right]\left[\begin{array}{c} v_{x}^{g}\\ q_{x}^{g} \end{array}\right].$$

All variables are non-dimensional and linearized. The matrix of Onsager coefficients reads [11,26]:

(A30) $$r_{\alpha \beta }=\left[\begin{array}{cc} \left(\frac{1}{\vartheta }-\frac{1}{2}\right)+\frac{1}{16} & \frac{1}{8}\\ \frac{1}{8} & \frac{1}{4} \end{array}\right].$$

The solutions based on (A30) are referred to as uncorrected NSF. A correction can be found in kinetic theory, which yields [11,26]:

(A31) $$r_{\alpha \beta ,corr}=\left[\begin{array}{cc} \frac{1}{\vartheta }-0.40044 & 0.126\\ 0.126 & 0.291 \end{array}\right].$$
